# Aconitate decarboxylase 1 participates in the control of pulmonary *Brucella* infection in mice

**DOI:** 10.1371/journal.ppat.1009887

**Published:** 2021-09-15

**Authors:** Aurore Demars, Armelle Vitali, Audrey Comein, Elodie Carlier, Abdulkader Azouz, Stanislas Goriely, Justine Smout, Véronique Flamand, Mégane Van Gysel, Johan Wouters, Jan Abendroth, Thomas E. Edwards, Arnaud Machelart, Eik Hoffmann, Priscille Brodin, Xavier De Bolle, Eric Muraille

**Affiliations:** 1 Unité de Recherche en Biologie des Microorganismes (URBM), NARILIS, University of Namur, Namur, Belgium; 2 Université Libre de Bruxelles, Institute for Medical Immunology, and ULB Center for Research in Immunology (U-CRI), Gosselies, Belgium; 3 Namur Medicine and Drug Innovation Center (NAMEDIC), Namur Research Institute for Life Sciences (Narilis), Department of Chemistry, Laboratoire de Chimie Biologique Structurale (CBS), Namur, Belgium; 4 UCB BioSciences, 7869 NE Day Road West Bainbridge Island, WA 98110 USA and Seattle Structural Genomics Center for Infectious Disease, Seattle, Washington, United States of America; 5 Université de Lille, CNRS, INSERM, CHU Lille, Institut Pasteur de Lille, U1019—UMR 9017—CIIL—Center for Infection and Immunity of Lille, Lille, France; 6 Université Libre de Bruxelles, Laboratoire de Parasitologie, and ULB Center for Research in Immunology (U-CRI), Gosselies, Belgium; University of California, Davis, UNITED STATES

## Abstract

Brucellosis is one of the most widespread bacterial zoonoses worldwide. Here, our aim was to identify the effector mechanisms controlling the early stages of intranasal infection with *Brucella* in C57BL/6 mice. During the first 48 hours of infection, alveolar macrophages (AMs) are the main cells infected in the lungs. Using RNA sequencing, we identified the aconitate decarboxylase 1 gene (*Acod1*; also known as Immune responsive gene 1), as one of the genes most upregulated in murine AMs in response to *B*. *melitensis* infection at 24 hours post-infection. Upregulation of *Acod1* was confirmed by RT-qPCR in lungs infected with *B*. *melitensis* and *B*. *abortus*. We observed that *Acod1*^-/-^ C57BL/6 mice display a higher bacterial load in their lungs than wild-type (wt) mice following *B*. *melitensis* or *B*. *abortus* infection, demonstrating that *Acod1* participates in the control of pulmonary *Brucella* infection. The ACOD1 enzyme is mostly produced in mitochondria of macrophages, and converts cis-aconitate, a metabolite in the Krebs cycle, into itaconate. Dimethyl itaconate (DMI), a chemically-modified membrane permeable form of itaconate, has a dose-dependent inhibitory effect on *Brucella* growth *in vitro*. Interestingly, structural analysis suggests the binding of itaconate into the binding site of *B*. *abortus* isocitrate lyase. DMI does not inhibit multiplication of the isocitrate lyase deletion mutant Δ*aceA B*. *abortus in vitro*. Finally, we observed that, unlike the wt strain, the Δ*aceA B*. *abortus* strain multiplies similarly in wt and *Acod1*^-/-^ C57BL/6 mice. These data suggest that bacterial isocitrate lyase might be a target of itaconate in AMs.

## Author summary

Brucellosis is one of the most widespread bacterial zoonoses worldwide. Here, our aim was to identify the effector mechanisms controlling the early stages of intranasal infection with *Brucella* in mice. During the first 48 hours of infection, alveolar macrophages (AMs) are the main cells infected in the lungs. Using RNA sequencing, a hypothesis-free approach, we identified the aconitate decarboxylase 1 gene (*Acod1*) as one of the genes most upregulated in murine AMs in response to *B*. *melitensis* infection. We observed that mice deficient for *Acod1* display a higher bacterial load in their lungs than wild-type (wt) mice following *B*. *melitensis* or *B*. *abortus* infection, demonstrating that *Acod1* participates in the control of pulmonary *Brucella* infection. We observed that, unlike the wt strain of *B*. *abortus*, the isocitrate lyase (ICL) deficient *B*. *abortus* strain multiplies similarly in wt and *Acod1* deficient mice, suggesting that bacterial ICL might be a target of *Acod1* in AMs. As the mouse ACOD1 enzyme is ∼80% identical in its amino acid sequence to the human ACOD1, the development of pharmacological agents that enhance ACOD1 function might help to control early stages of pulmonary *Brucella* infection.

## Introduction

*Brucella* spp. are facultative intracellular Gram-negative coccobacilli that infect mammals and cause brucellosis (reviewed in [1–4]). Human brucellosis is a zoonotic infection that is mainly transmitted through ingestion of food contaminated with *Brucella*. Aerosol contamination is also common following *Brucella*-induced abortions in farm animals or in butcher shops handling infected meats. Without prolonged antibiotics treatment, brucellosis causes a severe and debilitating chronic disease [1, 5].

The mouse is the experimental animal model most commonly used to study *Brucella* infection [6]. Following intranasal infection, *B*. *melitensis* multiplies in the lungs for several days before spreading to draining lymph nodes and then to the spleen where it persists for months [7]. Mice chronically infected with *B*. *melitensis* develop a protective memory response able to efficiently control a secondary *Brucella* infection [8]. However, this response is not able to eliminate bacteria that settled in CD11c^+^ splenic reservoir cells during primary infection [9], suggesting that splenic reservoir cells constitute a niche that hides *Brucella* from the Interferon(IFN)-γ-mediated protective immune response [10, 11]. Thus, in order to develop new therapeutic strategies against brucellosis, it would be very useful to identify the early mechanisms acting against *Brucella* in the lungs before it establishes its niche in the spleen.

Over the course of evolution, *Brucella* has acquired specific stealth strategies that allow it to reduce or interfere with its recognition by the immune system and neutralize immune effector mechanisms (reviewed in [3, 12]). Consequently, the inflammatory response against *Brucella* is particularly weak and difficult to detect. We showed previously that TCR-δ, TAP1, and IL-17RA deficiencies affect the early control of *B*. *melitensis* in the lungs (5 days post-infection) [8]. At high doses of infection, IL-1R and inflammasomes appear to be involved in early immune protective mechanisms against respiratory *B*. *abortus* infection (7 days post-infection) [13]. However, the precise effector immune mechanisms involved in early control of *Brucella* remain largely unknown.

Alveolar macrophages (AMs) are well known to act as first-line pulmonary immune sentinels and constitute the dominant immune cells in lungs at steady state [14]. In the first days of intranasal infection, CD11c^+^ F4/80^+^ AMs constitute the main pulmonary cells infected with *Brucella* [7, 15]. Their elimination increases the spread of *B*. *abortus* to the draining lymph nodes [15], suggesting that they are able to partially control *Brucella* multiplication. AMs perform a critical homeostatic role by clearing inhaled material from the airways and recycling pulmonary surfactant [14]. Via these steady-state functions, AMs express unique transcriptional and epigenetic profiles that are highly distinct from those of other tissue-resident macrophages [16]. It is therefore not surprising that *Brucella* was shown to induce weak IL-1β, IL-6, and TNF-α production in lung alveolar macrophages *in vitro* compared with peritoneal macrophages [17].

In the present study, to identify the mechanisms involved in the control of *Brucella* by AMs, we have chosen to use an unbiased approach involving RNA sequencing (RNAseq) on whole lung and purified AM samples from mice infected by the intranasal route with *B*. *melitensis*. We observed that the *Acod1/Irg1* gene, which codes for cis-aconitate decarboxylase 1 [18], is among the genes most overexpressed in response to infection and we demonstrate that *Acod1* participates in the direct control of *B*. *melitensis* and *B*. *abortus* multiplication in AMs.

## Results

### *Acod1* is among the genes most upregulated in alveolar macrophages from *B*. *melitensis* infected mice

**Fig 1:**
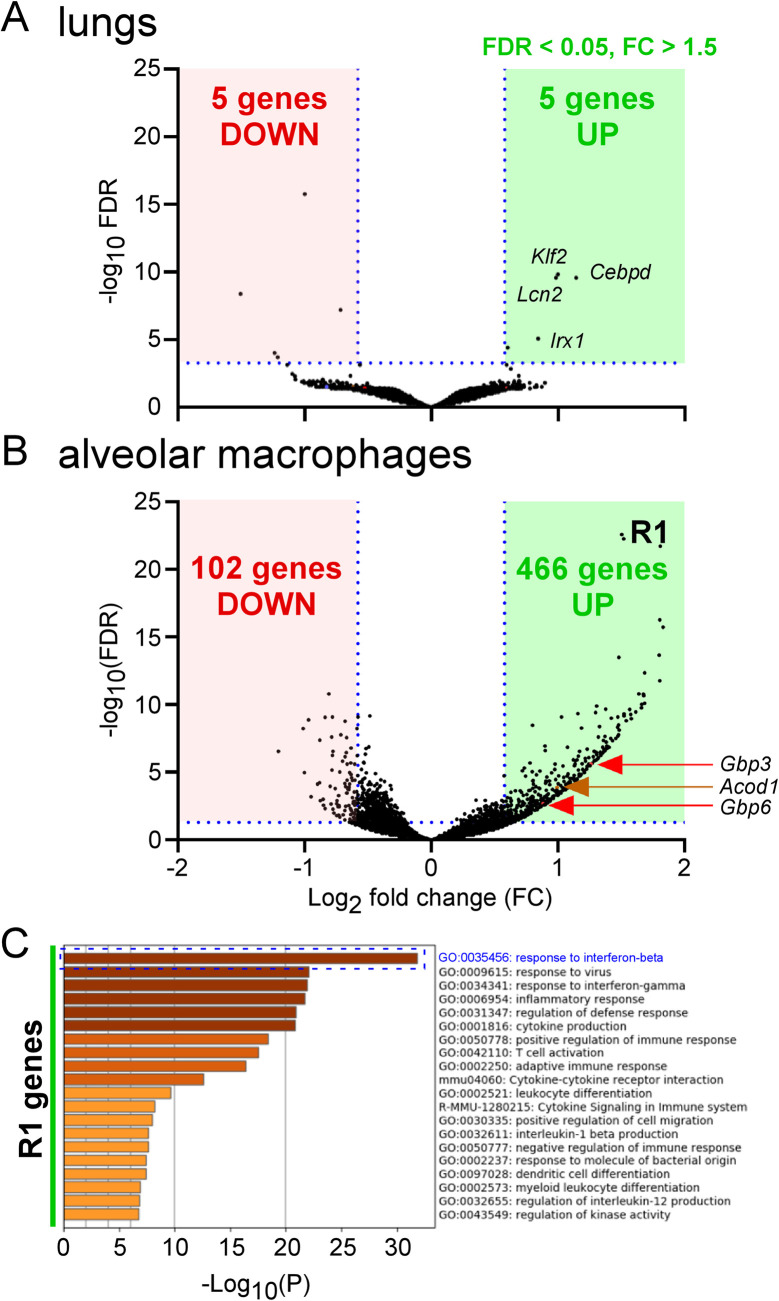
Identification of the most upregulated genes in lungs and alveolar macrophages from *B*. *melitensis*-infected mice. Wild-type C57BL/6 mice intranasally infected with 10^7^ CFU of wild-type *B*. *melitensis* 16M (n = 3), or receiving the same volume of PBS (naïve group) (n = 3) were sacrificed at 24 hours post-infection. Lungs were harvested and alveolar macrophages isolated as described in Materials and Methods. Then, RNA was extracted and sequenced with the Illumina system and Deseq2 analysis as described in the Materials and Methods. **(A)** Volcano plot of RNA-seq data from naïve versus infected lungs shows the adjusted p-value (false discovery rate, FDR -log10) versus fold change, FC (log2). The 5 upregulated genes are Lipocalin-2 (Lcn2), Krüppel-like Factor 2 (Klf2), Iroquois homeobox 1 (Irx1), CCAAT enhancer binding protein delta (Cebpd), Inter-Alpha-Trypsin Inhibitor Heavy Chain 4 (Itih4). The 5 downregulated genes are immunoglobulin heavy variable 5–6 (Ighv5-6), Elongation of very long chain fatty acids protein 6 (Elovl6), Immunoglobulin heavy variable 1–81 (Ighv1-81), Acetyl-CoA carboxylase 1 (Acaca), mitochondrially encoded tRNA asparagine (mt-Tn). **(B)** Volcano plot of RNA-seq data from naïve versus infected alveolar macrophages shows the adjusted p-value (false discovery rate, FDR -log10) versus fold change, FC (log2). The 466 genes with an FDR < 0.05 and FC > 1.5 are shown in the R1 green square. *Acod1* is the 178th most upregulated gene. **(C)** The R1 genes from alveolar macrophages were copy-pasted in Metascape (https://metascape.org) to perform a pathway enrichment analysis. Data are representative of two independent experiments.

In order to identify the immune effector mechanisms controlling the early multiplication of *Brucella melitensis* in the lungs, we compared gene expression in the lungs of *B*. *melitensis*-infected and PBS-treated wild-type (wt) C57BL/6 mice. Mice were infected with 10^7^ CFU of *B*. *melitensis* and sacrificed 24 hours later. Lungs were harvested and analyzed by RNA sequencing. The results of this analysis are presented in **S1 Data**. A volcano plot representation of these data shows that only a very low number of genes is significantly downregulated or upregulated during infection (**Fig 1A**). The 5 downregulated genes are immunoglobulin heavy variable 5–6 (*Ighv5-6*), Elongation of very long chain fatty acids protein 6 (*Elovl6*), Immunoglobulin heavy variable 1–81 (*Ighv1-81*), Acetyl-CoA carboxylase 1 (*Acaca*), mitochondrially encoded tRNA asparagine (*mt-Tn*). The 5 upregulated genes are Lipocalin-2 (*Lcn2*), that participates in the innate immune response against bacteria by sequestering iron [19], Krüppel-like Factor 2 (*Klf2*), Iroquois homeobox 1 (*Irx1*) and CCAAT enhancer binding protein delta (*Cebpd*) that are transcription factor genes and Inter-Alpha-Trypsin Inhibitor Heavy Chain 4 (*Itih4*).

We hypothesize that the early immune response against *Brucella* is not systemic in the lung and should be investigated at the cellular level, in the first cells infected with *Brucella*. Following intranasal infection, alveolar macrophages (AMs) constitute the main pulmonary cells infected with *B*. *melitensis* [7] *and B*. *abortus* [15] between 2 and 24 hours. In order to confirm this in our experimental model, we intranasally infected wt C57BL/6 mice with 10^7^ CFU of mCherry-*B*. *melitensis* stained with the fluorescent tracer eFluor670 which is used to visualize the cells infected with *B*. *melitensis* by flow cytometry [7]. We confirm that the main cells infected at 24 hours post-infection express the CD11c and Siglec-F markers (89.9% of all cells infected) and are therefore indeed AMs (**S1A Fig**). Then, in biosafety level 3 conditions, we purified the low-density lung cells expressing the CD11c marker in mice infected for 24 hours with 10^7^ CFU of eFluor670-stained mCherry-*B*. *melitensis*. This cell fraction was highly enriched in infected cells of the AM type and contained 73% CD11c^+^ eFluor^+^ cells (**S1B Fig**).

**Fig 2:**
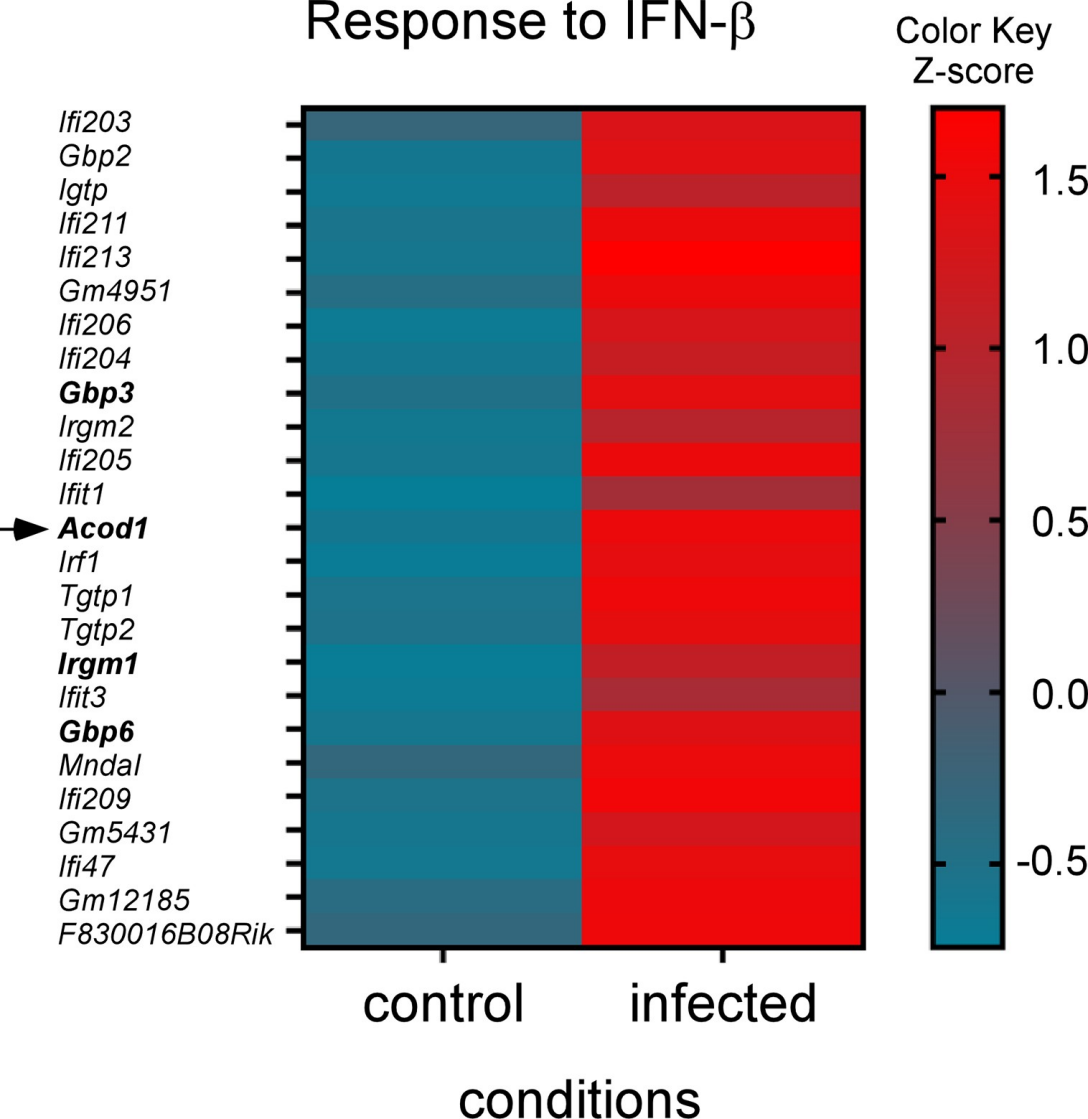
Response to interferon-beta genes” are upregulated at 24 hours post *B*. *melitensis* infection in mice. **“** The response to interferon-beta genes from the first pathway resulting from the Metascape analysis (see Fig 1C) were represented on a heatmap to see the expression of each gene in the pathway. The color scale indicates the Z score. RNAseq was performed at least two times and each sample was generated from a pool of at least 3 mice.

We compared the gene expression in an enriched AM fraction purified from the lungs of *B*. *melitensis*-infected and PBS-treated wt C57BL/6 mice. The results of this analysis are presented in **S2 Data**. A volcano plot representation of these data shows that 466 genes (R1 genes) were significantly upregulated in the infected mice (**Fig 1B**). We used the Metascape platform to perform a pathway enrichment analysis of these genes. The first signature identified was the response to interferon-beta (**Fig 1C**). The genes associated with this signature (R2 genes) were represented on a heatmap to see the expression of each gene in this pathway (**Fig 2**). Among them, some genes have been described to play an effector role in the innate immune response against intracellular pathogens. IFN-gamma-inducible GTP-binding protein *(Igtp)* participates in the disruption of *Toxoplasma gondii* vacuoles [20]. Guanylate-binding protein (*Gbp*)*3* and *Gbp6* regulate cell-autonomous immunity in macrophages by coordinating a potent oxidative and vesicular trafficking program to protect the host from intracellular bacteria [21]. The implication of GBP proteins in the Stimulator of Interferon Genes (STING)-dependent type I interferon response against *B*. *abortus* has already been described [22]. One very interesting candidate is the *Acod1*/*Irg1* gene which has been described to act as a direct effector against intracellular bacteria as well as a regulator of the inflammatory response (reviewed in [23]).

### *Acod1* deficiency induces a lack of *Brucella* control in the lungs

**Fig 3:**
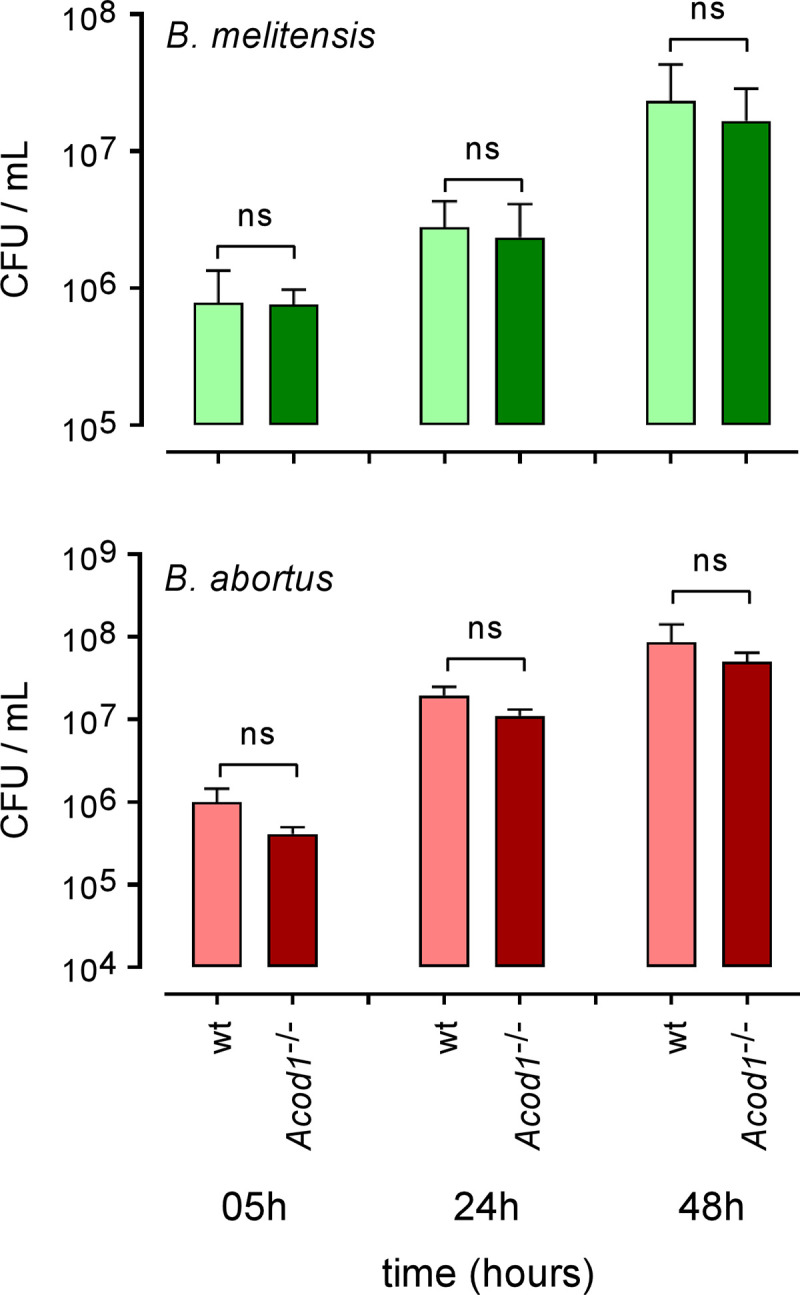
*Acod1* is dispensable for Bone marrow derived macrophages control of *Brucella* infection. Bone marrow derived macrophages from wild type and *Acod1*^-/-^ C57BL/6 mice were infected with *B*. *melitensis* or *B*. *abortus* at an MOI of 50:1, as indicated in Materials and Methods. At various timepoints after infection (5h, 24h and 48h), intracellular CFU levels were measured. Error bars depict S.D. of the mean. Data are representative of 4 independent experiments. ns = non-significant.

**Fig 4:**
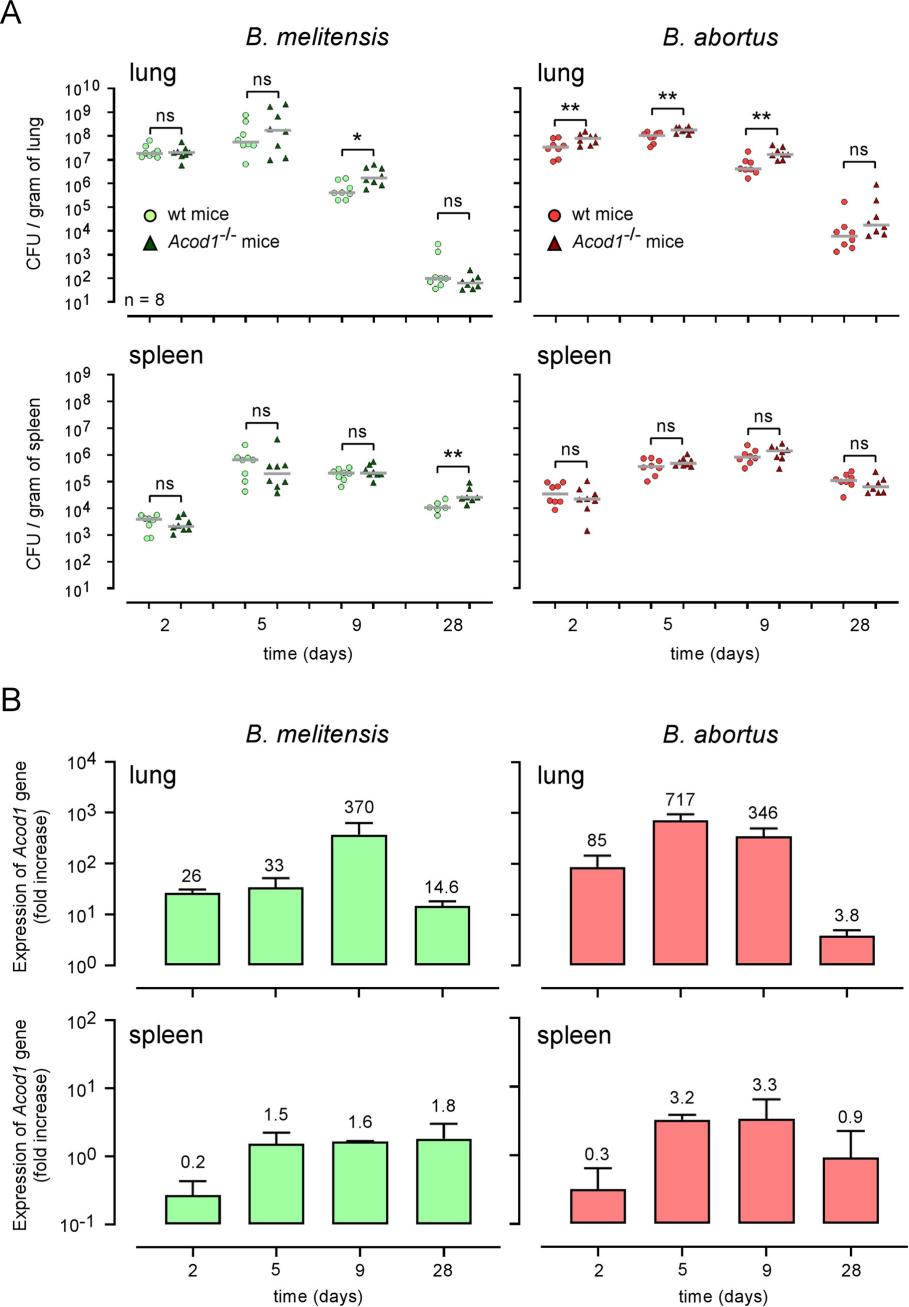
*Acod1* gene expression is correlated to *Brucella* control in the lungs. **(A)** Wild-type and *Acod1*^-/-^ C57BL/6 mice were intranasally infected with 10^7^ CFU of wild-type *B*. *melitensis* 16M or *B*. *abortus* 2308, as indicated. At 2-, 5-, 9- and 28-days post-infection, lungs and spleen were harvested and CFU were counted. Each point represents one mouse. n = number of mice used. Grey bar represents the mean. Significant differences between the indicated groups are marked with asterisks: *p < 0.05, **p < 0.01. ns = non-significant. Data are representative of at least 3 independent experiments. **(B)** Wild-type C57BL/6 mice were intranasally infected with 10^8^ CFU of wild-type *B*. *melitensis* 16M or *B*. *abortus* 2308, as indicated. At 2-, 5-, 9- and 28-days post-infection, lungs and spleen were harvested and RNA was extracted. After reverse-transcription into cDNA, qRT-PCR was performed with *Acod1* primers. At least 6 mice were pooled for each condition. The data are representative of 3 independent experiments. The mean fold change number is indicated at the top of the histogram bar.

As a first attempt to establish a role of ACOD1 in the control of intracellular *Brucella* multiplication, we infected *in vitro* bone marrow derived macrophages (BMDMs) from wild-type or *Acod1*^-/-^ C57BL/6 mice with *B*. *melitensis* or *B*. *abortus*. We observed that the multiplication of both *Brucella* species is not significantly affected by *Acod1* deficiency in BMDMs (**Fig 3**). Others [24] have shown that during *M*. *tuberculosis* infection *Acod1* deficiency affects the control of infection *in vivo* but not *in vitro*. Thus, to formally determine whether or not ACOD1 participates in the control of *Brucella* infection, we compared the CFU count of bacteria in the lungs and spleen of wt and *Acod1*^-/-^ C57BL/6 mice intranasally infected with 10^7^ CFU of wt *B*. *melitensis* or *B*. *abortus* (**Fig 4A**). We observed that *Acod1*^-/-^ mice infected with *B*. *melitensis* only displayed significantly enhanced CFU at 9 days post-infection in the lungs, compared to wt mice. In contrast, CFU counts were significantly enhanced at 2-, 5- and 9-days post-infection in *Acod1*^-/-^ mice infected with *B*. *abortus*. This difference may be the consequence of the lower induction of *Acod1* in response to infection with *B*. *melitensis* (**Fig 4B**). In agreement, *Acod1* deficiency had only a minimal impact on the course of *B*. *melitensis* and *B*. *abortus* in the spleen (**Figs 4A** and S2) where no *Acod1* expression was detected (**Fig 4B**).

Finally, we also compared the CFU count of bacteria in the spleen of wt and *Acod1*^-/-^ C57BL/6 mice intraperitoneally infected with 10^7^ CFU of wt *B*. *abortus* (**S3 Fig**). We observed that *Acod1* deficiency does not affect the control of *B*. *abortus* in spleen, which suggests that *Acod1* would only play a role in specific organs such as lungs.

### Enhanced susceptibility of *Brucella*-infected *Acod1*^-/-^ mice is not associated with higher inflammation in the lungs

*Acod1* deficiency has been associated with a lack of inflammatory control in several models of bacterial [24] and viral [25] infection in mice. For example, *Acod1*^-/-^ but not wt C57BL/6 mice intranasally infected with *M*. *tuberculosis* succumbed rapidly, and mortality was associated with increased infection, neutrophilia and production of inflammatory cytokines [24]. Depletion of neutrophils enhances survival of *M*. *tuberculosis*-infected *Acod1*^−/−^ mice.

**Fig 5:**
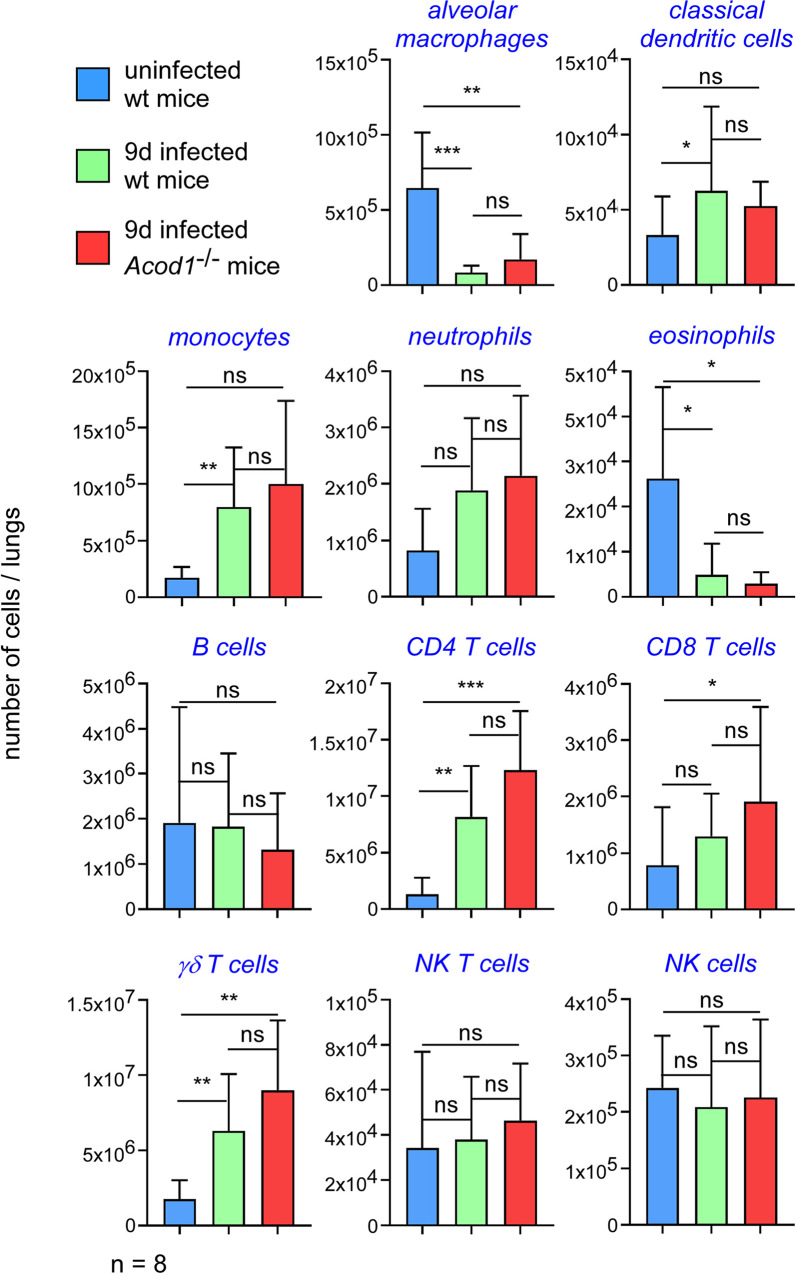
Enhanced susceptibility of *B*. *abortus* infected *Acod1*^*-/-*^ mice is not associated to different cell recruitment in the lungs. Wild-type and *Acod1*^-/-^ C57BL/6 mice were intranasally infected with 10^7^ CFU of wild-type mCherry expressing *B*. *abortus* 2308. At 9 days post-infection, mice were sacrificed and lungs were harvested. Lung cells were isolated and then analyzed by flow cytometry as described in S6 Fig. Uninfected mice served as controls. Significant differences between the indicated groups are marked with asterisks: *p < 0.05, **p < 0.01, ***p < 0.001. ns = non-significant. The cells were counted out of the total number of cells by organ. Data represent the number of different cell populations per lung from individual mice. 3 and 6 mice were used for the control or infected condition, respectively. These data are representative of 2 independent experiments.

**Fig 6:**
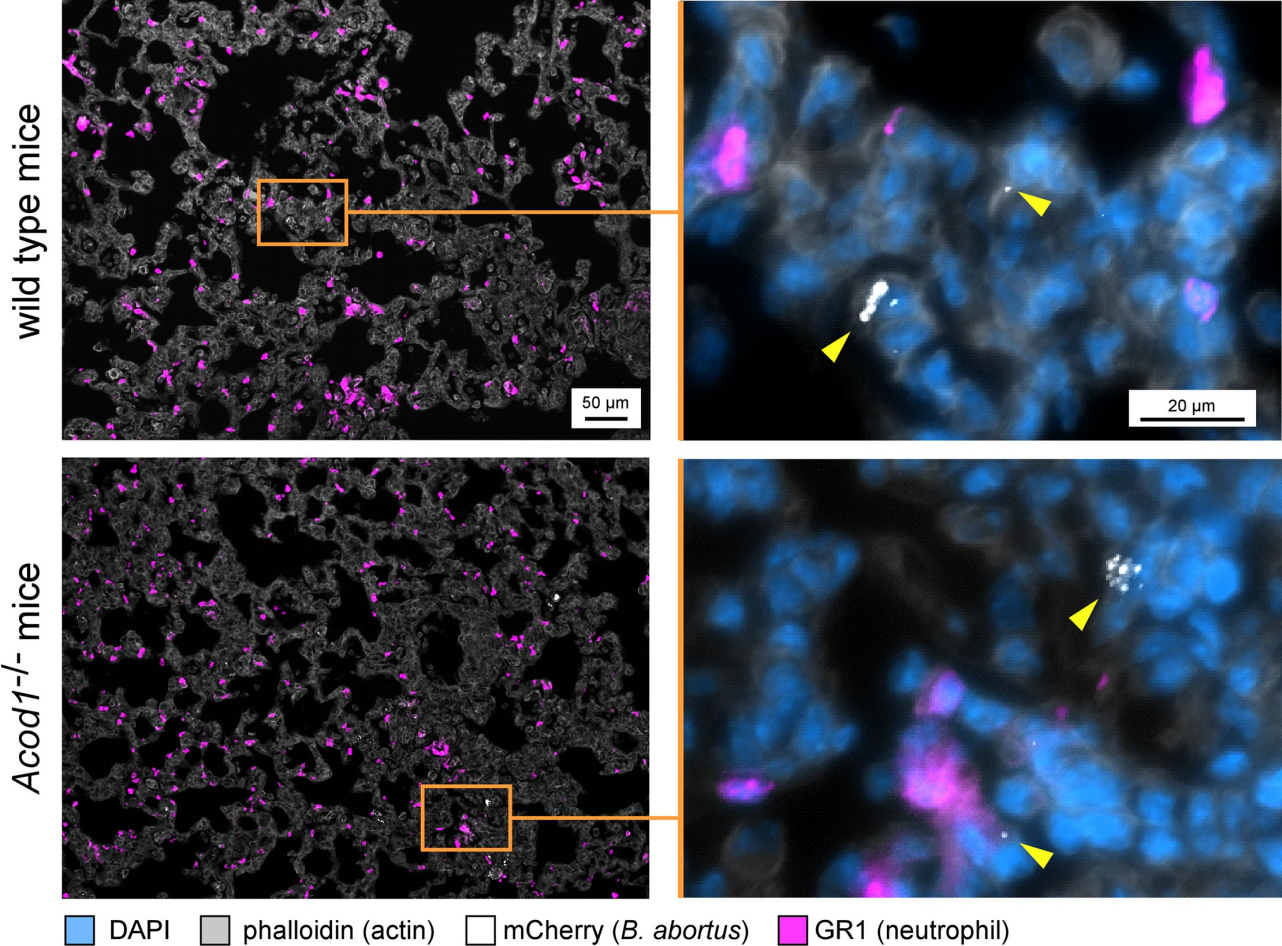
Enhanced susceptibility of *B*. *abortus* infected *Acod1*^*-/-*^ mice is not associated to higher cell recruitment in the lungs. Wild-type and *Acod1*^-/-^ C57BL/6 mice were intranasally infected with 10^7^ CFU of wild-type mCherry expressing *B*. *abortus* 2308. At 9 days post-infection, mice were sacrificed and lungs were harvested. Lungs were fixed and embedded. 5 μm-thick slides of were made with a cryostat. Slides were stained and analyzed by fluorescent microscopy for the expression of DAPI, phalloidin, mCherry and neutrophil (GR1) marker. The yellow triangles indicate the presence of *B*. *abortus* expressing the mCherry fluorescent tracer. These images are representative of at least 2 independent experiments on at least 3 mice per group.

**Fig 7:**
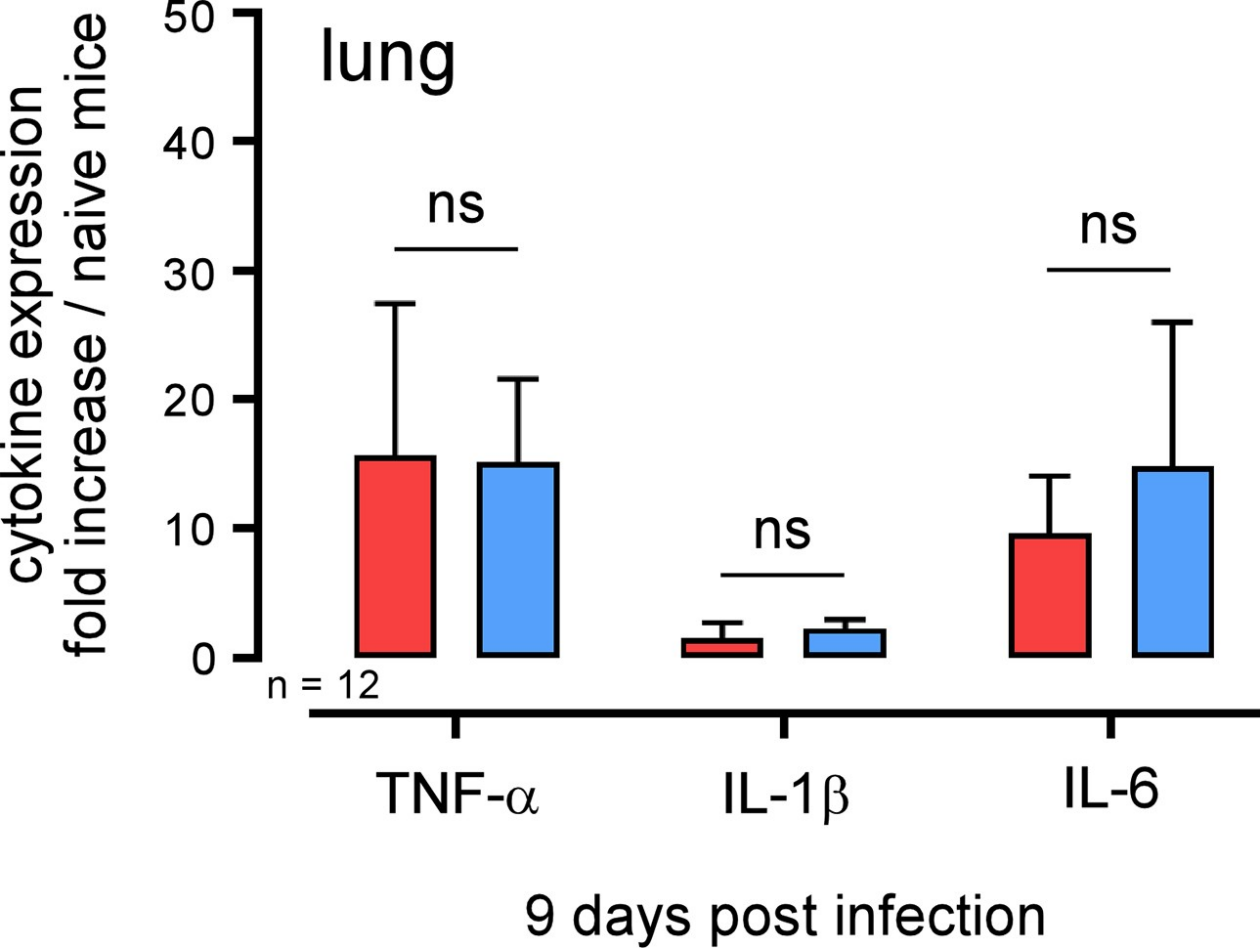
Enhanced susceptibility of *B*. *abortus* infected *Acod1*^*-/-*^ mice is not associated with higher pro-inflammatory cytokine expression in the lungs. Wild-type (red) and *Acod1*^-/-^ (blue) C57BL/6 mice were intranasally infected with 10^7^ CFU of wild-type mCherry expressing *B*. *abortus* 2308. At 9 days post infection, mice were sacrificed, lungs were collected, RNA was extracted and qRT-PCR was performed. TNF-α, IL-1β, and IL-6 expression levels were analyzed. *Tbp* gene was used as negative control, and RNA from naïve mice was used as the standard condition. Data show the fold increase of RNA expression for the indicated cytokine from infected mice compared to control mice. At least 6 mice were pooled for each condition. The data are representative of 3 independent experiments. ns = non-significant.

In order to determine whether *Acod1* tempered *Brucella*-induced inflammation in our experimental model or not, we analyzed the cellular recruitment induced by *B*. *abortus* in the lungs of infected mice. Wt and *Acod1*^-/-^ C57BL/6 mice were infected with 10^7^ CFU of *B*. *abortus* and sacrificed 9 days post-infection. The lungs were removed and analyzed by flow cytometry and fluorescence microscopy. The cytometric analysis shows that *B*. *abortus* infection induced a significant increase in the number of monocytes, CD4^+^T cells and γδT cells and a significant decrease of alveolar macrophages and eosinophils number (**Figs 5** and S4 for gating strategy). However, these changes in cellular composition of the lungs did not differ significantly between wt and *Acod1*^-/-^ mice. Similarly, histological analysis showed no clear differences between *B*. *abortus* infected lungs of wild-type and *Acod1*^-/-^ mice in terms of recruitment of neutrophils and we did not observe clusters of neutrophils (GR1^+^ cells) around cells infected with *B*. *abortus* in the lungs (**Fig 6**). Finally, measurement of the expression of the proinflammatory cytokines TNF-α, IL-1β and IL-6 in these mice by RT-qPCR showed that these cytokines were indeed induced by *B*. *abortus* infection but were not significantly more expressed in the *Acod1*^-/-^ mice than in the wt mice (**Fig 7**). On the whole, our data show that *Acod1* deficiency did not lead to a detectable increase of inflammation in mice infected with *B*. *abortus*.

### In neutral pH conditions, dimethyl itaconate and 4-octyl itaconate, but not itaconate, inhibit *Brucella* growth in culture

**Fig 8:**
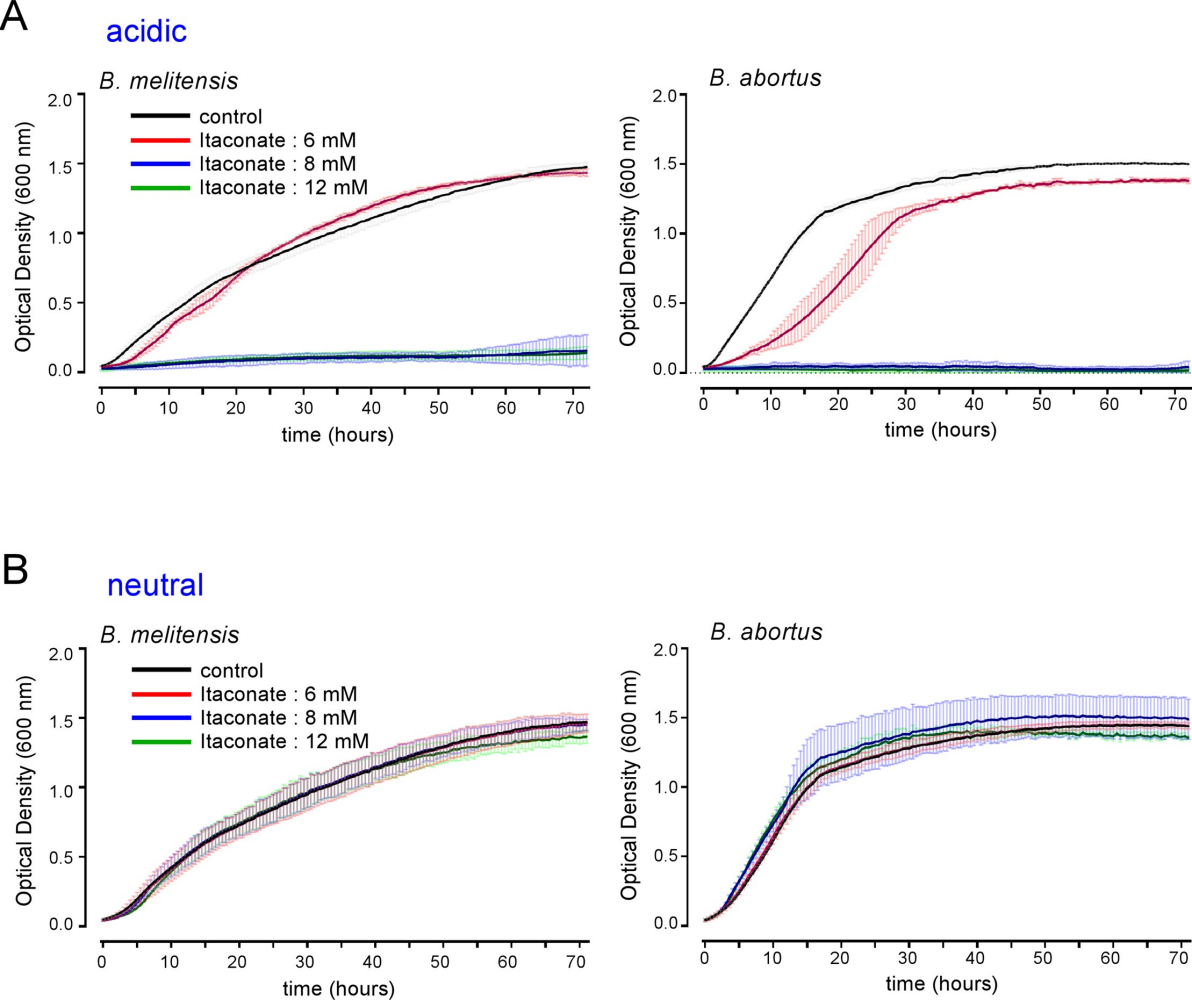
At neutral pH, itaconate does not inhibit *Brucella* growth *in vitro*. Comparison of the impact of different concentrations of itaconate on the growth of wild-type *B*. *melitensis* (left-hand panel) or *B*. *abortus* (right-hand panel) in rich medium (2YT). **A.** acidic itaconate (pH ≃ 3.6). **B**. neutralized itaconate solution (pH = 7.0). The bacteria were grown for 72 hours at 37°C and the OD was measured every 30 min in a Bioscreen system. The standard deviation was obtained from three independent experiments.

**Fig 9:**
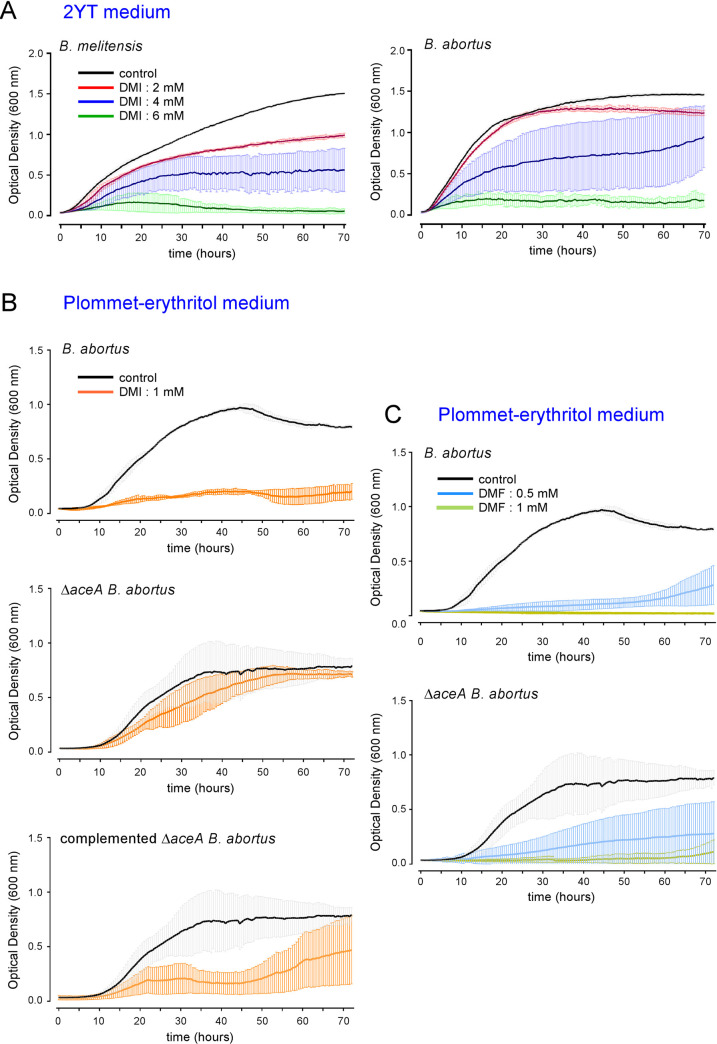
The multiplication of *Brucella in vitro* is inhibited by dimethyl itaconate via an isocitrate lyase-dependent mechanism. **(A)** Comparison of the impact of different concentrations of dimethyl itaconate (DMI) on the growth of wild-type *B*. *melitensis* (left panel) or *B*. *abortus* (right panel) in rich medium (2YT). **(B)** Comparison of the impact of 1 mM of DMI on the growth of wild-type, Δ*aceA* and Δ*aceA-*complemented *B*. *abortus* in poor medium (Plommet-Erythritol). (**C**) Dimethyl fumarate (DMF) was used as control, in the same conditions as (B). The bacteria were grown for 72 hours at 37°C. The OD was measured every 30 min in a Bioscreen system. Data points represent means and standard deviations of three independent experiments.

The *Acod1* gene codes for a cis-aconitate decarboxylase that converts cis-aconitate into itaconate [18]. The itaconate concentration reached 5 mM in mouse Bone Marrow-Derived Macrophages (BMDMs) after LPS stimulation [26]. It has been reported that itaconate could directly inhibit the *in vitro* multiplication of many bacteria such as *Salmonella enterica*, *Mycobacterium tuberculosis* [18], *Legionella pneumophila*, *Staphylococcus aureus* and *Acinetobacter baumannii* [27]. As expected, we found that itaconate completely inhibited the *in vitro* growth of *B*. *melitensis* and *B*. *abortus* in rich acidic medium (2YT, pH ~3.5) at concentration (8–12 mM) close to those measured in stimulated BMDMs (**Fig 8A**). Surprisingly, at neutral pH in 2YT, itaconate did not seem to have any significant effect on *Brucella* multiplication in *vitro*, regardless of the species (**Fig 8B**). This may have been due to the inability of itaconate to cross the bacterial membrane because of its charged nature. *In vivo*, after phagocytosis, *Brucella* is thought to be at acidic pH in the phagolysosome of the host cell, thus increasing the possibility of increased membrane permeability. Therefore, itaconate might be able to enter *Brucella* in physiological conditions *in vivo* and exert its potentially inhibitory effect. To validate this hypothesis, we tested the impact of different concentrations of dimethyl itaconate (DMI) [28], a membrane-permeable non-ionic form of itaconate, on the multiplication of *B*. *melitensis* and *B*. *abortus in vitro* at neutral pH. We observed that DMI inhibits the growth of both *Brucella* species in a dose-dependent manner in rich medium (2YT) (**Fig 9A**), suggesting that itaconate is able to specifically affect *Brucella* when it can cross its membrane. Similar, results were obtained with 4-octyl itaconate [26], another membrane-permeable non-ionic form of itaconate (**S5 Fig**).

*B*. *abortus* bacteria incubated for 24 hours in the presence of 10 mM DMI in 2YT, washed and then cultured again in the absence of DMI showed 100 times less CFU than control bacteria not treated with DMI (**S6 Fig**), which demonstrates that DMI is bactericidal for *B*. *abortus*.

### Inhibition of *Brucella* growth in culture by dimethyl itaconate is isocitrate lyase-dependent

Itaconate has been described to act via the bacterial enzyme isocitrate lyase (ICL) [18] which is part of the glyoxylate shunt that is exclusively found in prokaryotes, lower eukaryotes, and plants. This alternative shunt is activated in response to nutrient deprivation, a condition encountered by bacteria in phagolysosomes [29].

**Fig 10:**
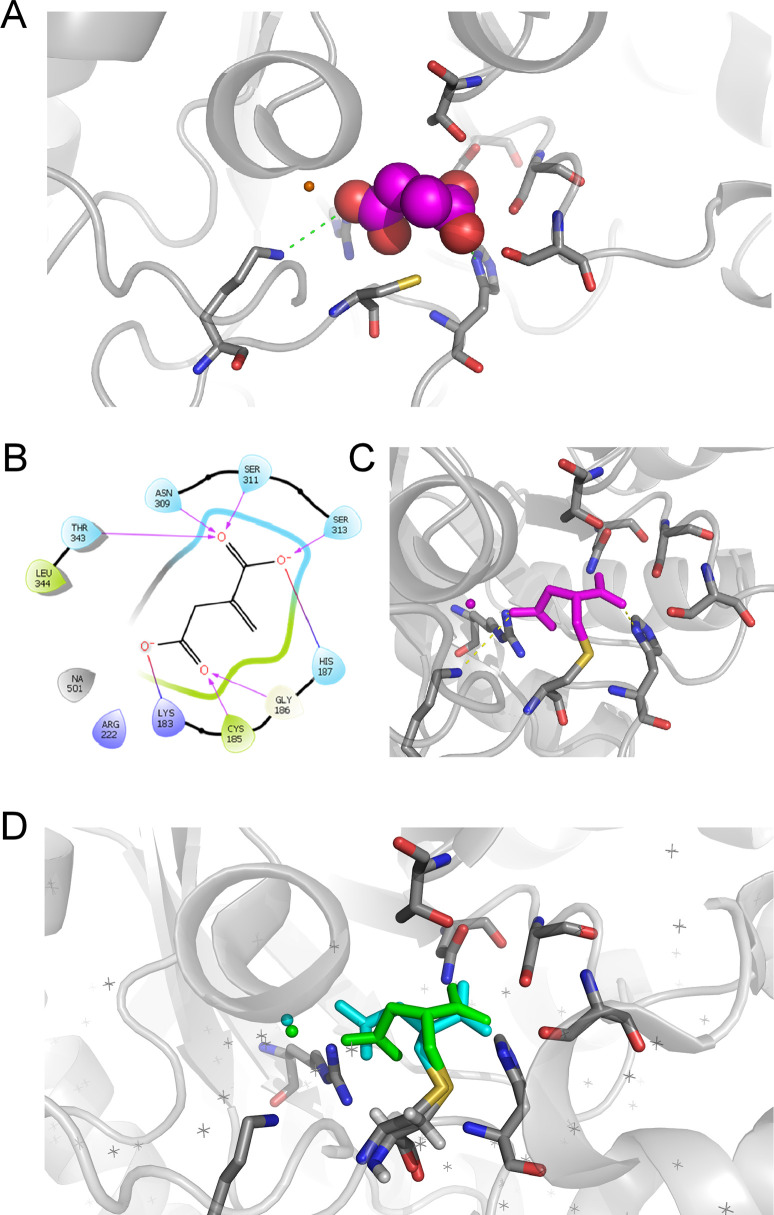
Docking and crystallography supports binding of itaconate into the binding site of isocitrate lyase. **(A)** Docking pose of itaconate (magenta) into the crystal structure of isocitrate lyase from *Brucella abortus* (PDB entry 3OQ8) suggests a good binding of this compound in the active site of the protein. **(B)** The ligand in stabilized in the enzyme by salt bridges involving the two carboxylate groups of itaconate and residues Lys183, Arg222, and His187. A network of Hydrogen bonds further stabilizes the ligand inside the enzyme. In this geometry, Cys185 is well positioned to react with the reactive double bond of itaconate. **(C)** Docking suggests that a covalent adduct (magenta) can be formed by reaction of the lateral thiol group of Cys185 (yellow sulfur atom) and the carbon atom of the reactive double bond of itaconate. **(D)** The experimental crystal structure (blue) of the covalent complex of itaconate with *B*. *abortus* isocitrate lyase (PDB entry 7RBX) confirms the docking pose (green).

Starting from the crystal structure of isocitrate lyase from *B*. *abortus* in complex with malonate (Protein Data Bank (PDB) entry 3OQ8) docking of itaconate has been performed and suggests a good binding of this compound in the active site of the protein (**Fig 10A**). The ligand is stabilized in the enzyme by salt bridges involving the two carboxylate groups of itaconate and residues Lys183, Arg222, and His187 (**Fig 10B**). A network of Hydrogen bonds further stabilizes the ligand inside the enzyme (**Fig 10C**). Interestingly, this docking pose places the carbon atom of the reactive double bond of itaconate in close proximity with the lateral chain of Cys185, suggesting a possible nucleophilic attack by the thiol of this residue leading to a covalent adduct between isocitrate lyase and itaconate. This docking pose has been experimentally confirmed by crystallography (PDB entry 7RBX) that unambiguously shows a covalent adduct between itaconate and Cys185 in *B*. *abortus* isocitrate lyase (**Fig 10D**).

We compared the effect of DMI on the growth of a wt strain and an ICL-deficient strain (Δ*aceA*) of *B abortus* in a defined minimal medium for *Brucella*, the Plommet-Erythritol medium [30]. We observed that the growth of the Δ*aceA* strain was much less affected by DMI than the wt strain (**Fig 9B**). Complementation of the Δ*aceA* mutant with a plasmid encoding ICL renders it sensitive to DMI, which demonstrates that itaconate acts via ICL to inhibit the multiplication of *B*. *abortus* under our experimental conditions. In contrast, dimethyl fumarate (DMF), which has been described to inhibit the growth of *E*. *coli* [31], reduces the growth of wt and Δ*aceA B*. *abortus* similarly (**Fig 9C**), suggesting that DMI is specifically responsible for the ICL-dependent inhibition of *B*. *abortus* growth.

DMI treatment induces significant morphological changes in *Brucella*. Transmission electron microscopy (TEM) analysis shows that wt *B*. *abortus* treated for 24 hours with 1 mM DMI present significant thickening of the envelope (**S7 Fig**). However, these alterations are also observed with DMI-treated Δ*aceA B*. *abortus*, demonstrating that these envelope defects do not correlate with the growth inhibition effect of DMI.

### *Acod1* deficiency does not affect the growth of *ΔaceA B*. *abortus in vivo*

**Fig 11:**
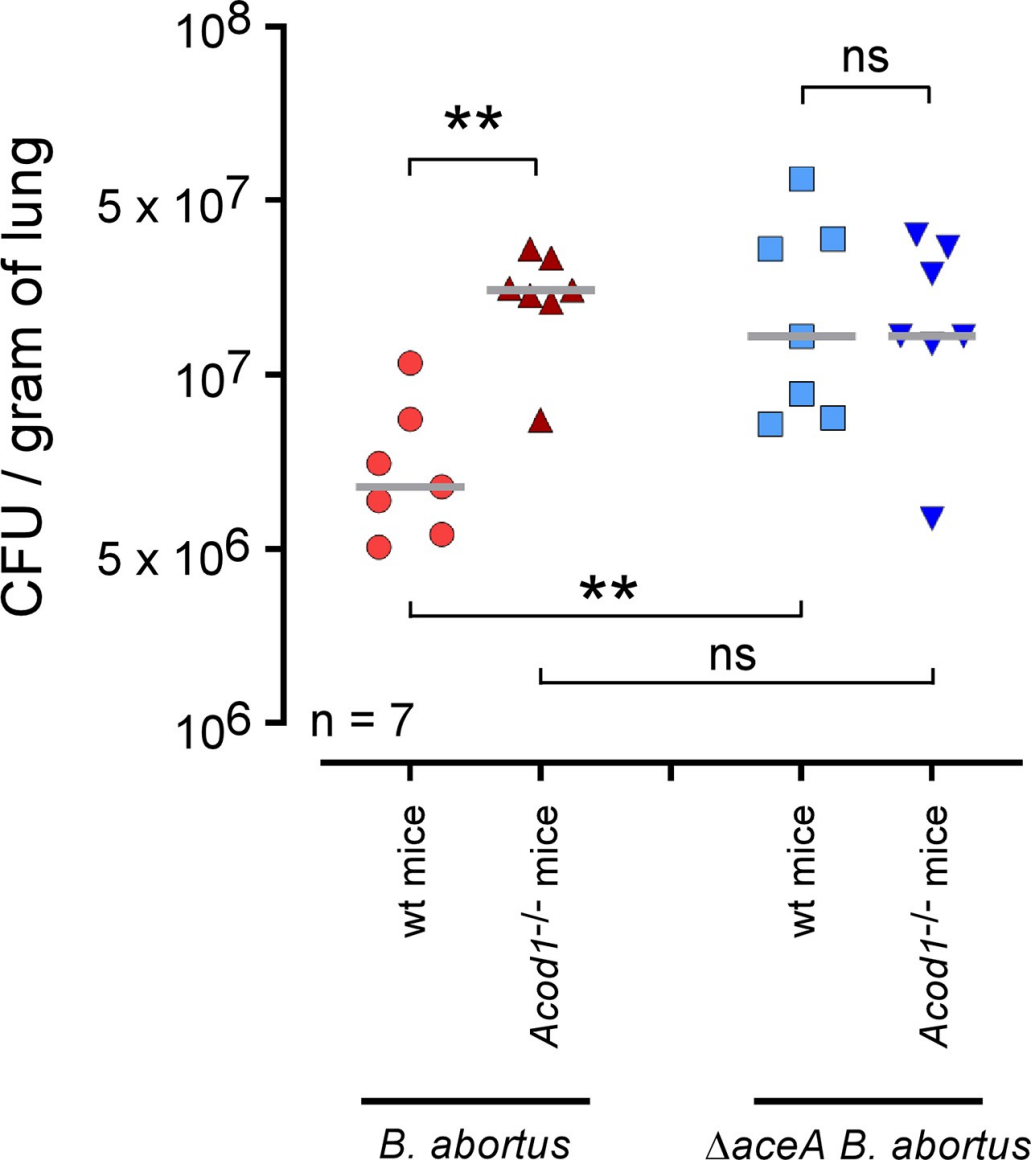
*Acod1* deficiency does not affect the growth of Δ*aceA B*. *abortus in vivo*. Wild-type and *Acod1*^-/-^ C57BL/6 mice were intranasally infected with 10^7^ CFU of wild-type or Δ*aceA* mCherry expressing *B*. *abortus* 2308, as indicated. At 9 days post infection, mice were sacrificed, lungs were harvested and CFU were counted. Each point represents one mouse, n = number of mice used for each condition. Grey bar represents the mean. Significant differences between the indicated groups are marked with asterisks: **p < 0.01. ns = non-significant. The data are representative of at least 3 independent experiments.

Finally, to determine whether *Acod1*-dependent control of *Brucella* infection *in vivo* is acting via bacterial ICL, wt and *Acod1*^-/-^ C57BL/6 mice were intranasally infected with 10^7^ CFU of wt or Δ*aceA B*. *abortus* and sacrificed 9 days post-infection. In striking contrast with the wt strain of *B*. *abortus*, CFU analysis of lungs from infected mice showed that growth of the Δ*aceA* strain was not affected by *Acod1* deficiency (**Fig 11**), demonstrating that ACOD1 control of *B*. *abortus* in the lungs is indeed dependent on the expression of ICL by *Brucella*. In addition, the fact that the CFU in *Acod1*^-/-^ mice infected with wt and Δ*aceA B*. *abortus* were comparable is consistent with the hypothesis that ICL is the main target of ACOD1.

## Discussion

Mucosal surfaces are portals of entry for the vast majority of pathogens. In the case of brucellosis, natural infections take place mainly via the intestinal [32] and respiratory tracts [33]. Despite this, little is known about the immune mechanisms controlling *Brucella* at the mucosal level. We have shown previously that the early multiplication of *B*. *melitensis* in the lungs of infected mice is controlled by the immune system. The comparison of mice genetically deficient for key elements of the innate and adaptive immune response demonstrated that γδ T lymphocytes, CD8 T lymphocytes as well as pathways dependent on the IL-17RA receptor were involved [8]. However, the precise mechanisms directly controlling the proliferation of *Brucella* in the lungs remain largely unknown. In order to identify them, we used an unbiased approach that involved performing RNA sequencing of the whole lungs as well as the main cells infected with *Brucella*, the alveolar macrophages.

Intranasal *B*. *melitensis* infection does not appear to induce any detectable systemic response in the lungs at 24 hours post-infection, even at a dose of 10^7^ CFU. RNA sequencing analysis shows that only 5 genes display a significant increase in expression in the lungs from infected wild type C57BL/6 mice. This contrasts sharply with the increase in the expression of 1526 genes observed in the lungs of wild type C57BL/6 mice after intranasal infection with 10^6^ CFU of *Legionella pneumophilia* [27], but fits well with the stealth pathogen profile of *Brucella* [3, 34]. However, analysis of the RNA profile of purified alveolar macrophages allowed us to identify 466 genes whose expression was significantly increased compared to uninfected controls, which shows that *Brucella* induces an immune response within infected cells. A clustering analysis carried out using Metascape led us to select several genes involved in the anti-bacterial immune responses induced by type 1 interferon, such as *Igtp*, *Gbp3*, *Gbp6* and *Acod1*. Among these, *Acod1* has been described to play dual roles in immunity and diseases (for a review see [23]). We therefore chose to explore its role in the pulmonary immune response against *Brucella*.

The *Acod1* gene was originally identified in 1995 as a 2.3-kb cDNA from a library synthesized from mRNA isolated from a murine macrophage cell line after LPS stimulation [35]. It is markedly upregulated in response to pathogen-associated molecular patterns, such as LPS and CpG and inflammatory cytokines, such as type I interferon and tumor necrosis factor. The ACOD1 enzyme catalyzes itaconate metabolite production by cis-aconitate decarboxylation and is mainly found in mitochondria of myeloid cells.

Itaconate is well described to downregulate pro-inflammatory cytokine and reactive oxygen species production by multiple mechanisms [23]. *Acod1* expression has been reported to temper inflammation and prevent immune-related pathology during *Mycobacterium tuberculosis* [24] and Respiratory Syncytial Virus [25] infections in mice. In striking contrast, in our *Brucella* infection model, we showed that the absence of *Acod1* does not significantly increase proinflammatory cytokine production or cellular recruitment in the lungs of infected mice, despite a significant increase in the bacterial load in the lungs. Again, this must be the consequence of the stealth pathogen profile of *Brucella*.

Itaconate is also known for its ability to directly inhibit the multiplication of several bacteria *in vitro* [18] and *in vivo* [27]. We have shown that two membrane-permeable forms of itaconate, DMI and 4OI, fully inhibit *Brucella* multiplication *in vitro*. However, in mice, *Acod1* deficiency has only a moderate effect on the multiplication of *Brucella*. This can be explained by the existence of multiple effector mechanisms controlling *Brucella in vivo*. For example, we [36, 37] and others [38] have shown that a deficiency in inducible NO synthase (iNOS) increases susceptibility to *Brucella* infection in mice. In addition, GBP proteins, whose expression is also found to be increased in *Brucella*-infected alveolar macrophages, have been described as participating in the control of *Brucella in vitro* and *in vivo* [22]. This could explain that the deficiency of a single specific effector mechanism cannot lead to the collapse of the entire protective response against *Brucella*. Surprisingly, although *B*. *melitensis* and *B*. *abortus* display the same sensitivity to DMI and 4OI in vitro, *Acod1* deficiency increases susceptibility to *B*. *abortus* much more than to *B*. *melitensis* in lungs. This difference can be correlated to the earlier and stronger induction of *Acod1* expression in the lungs by *B*. *abortus* than by *B*. *melitensis* following intranasal infection. This could be explained by the slightly higher CFU level of *B*. *abortus* compared to *B*. *melitensis* in the lungs of wt mice. Another non-exclusive possibility would be that genes involved in itaconate degradation are differentially regulated between *B*. *abortus* and *B*. *melitensis*. Indeed, homologs of previously identified genes involved in itaconate degradation in *Yersinia pestis* [ref PMID: 24657929], namely *ripA* (ict), *ripB* (ich) and *ripC* (ccl), have homologs in *B*. *abortus* and *B*. *melitensis* (BAB1_1077/BMEI0928, BAB1_1554/BMEI0478 and BAB2_0355/BMEII0413 respectively). In this scenario, *B*. *melitensis* would more efficiently degrade itaconate than *B*. *abortus* in this model of infection.

During the submission of our article, a study by Lacey *et al*. [39] showing that *Acod1* deficiency led to a slightly but significant increase CFU count of *B*. *melitensis* in the lung but not the spleen from intranasally infected mice has been published. This result fully confirms our observations in the mouse model. The authors also report that *Acod1* deficiency does not affect the multiplication of *B*. *melitensis* in BMDMs, which confirms that the *in vitro* infection of BMDM cannot be used as a predictive model for the infection of alveolar macrophages *in vivo*.

Itaconate is known to inhibit bacterial multiplication via isocitrate lyase (ICL), the key enzyme of the glyoxylate shunt, a two-step metabolic pathway that acts as an alternative shunt to the tricarboxylic acid cycle and is essential for bacterial growth under specific conditions. In *Mycobacterium tuberculosis*, ICL also exhibits additional methyl-ICL activity that is required for the detoxification of propionyl-CoA through the 2-methylcitrate cycle [18]. No attenuation of the ICL-deficient *B*. *abortus* strain, Δ*aceA*, was reported in BALB/c mice [40]. We showed that Δ*aceA B*. *abortus* is more resistant to itaconate inhibition *in vitro* than the wt bacterial strain and displays similar multiplication in wt and *Acod1*^*-/-*^ mice. On the whole, our results support a model in which the ACOD1 enzyme, via the production of itaconate, restricts *Brucella* replication in murine alveolar macrophages through a bacterial ICL-dependent mechanism. This hypothesis is supported, in part, by docking studies that place the carbon atom of the reactive double bond of itaconate in close proximity with the lateral chain of Cys185 in *B. abortus* isocitrate lyase, potentially leading to a nucleophilic attack by the thiol and formation of a covalent adduct between isocitrate lyase and itaconate. This docking pose has been experimentally confirmed by crystallography (PDB entry 7RBX) that unambiguously shows a covalent adduct between itaconate and Cys185 in *Brucella abortus* isocitrate lyase. However, *aceA* deficiency does not prevent or reduce *Brucella* multiplication in the lungs, demonstrating that ICL is not essential to *Brucella* multiplication in this condition. This suggests that *Acod1*-mediated ICL-dependent inhibition of *Brucella* multiplication in our murine infection model is not explained by a simple blockage of the glyoxylate shunt or by the toxic accumulation of propionyl-CoA. The precise mechanism of *Brucella* growth inhibition by itaconate therefore remains to be elucidated. It is interesting to remark that DMI also induced massive disorganization of the bacterial envelope, independently of ICL. One possible mechanism for this effect could be the inhibition of several L,D-transpeptidases, which are enzymes that crosslink the peptide stems of peptidoglycan. These enzymes have a cysteine in their active site and itaconate is known to alkylate this residue.

Our results demonstrate that *Acod1* plays very different roles depending on the bacterial species. Its ability to regulate inflammation does not seem to be exercised against stealthy bacteria such as *Brucella*, although it is essential for the control of neutrophil-mediated immune-related pathology during *M*. *tuberculosis* infection [24]. Itaconate is able to inhibit bacterial multiplication through a variety of pathways, which might be reflective of metabolic differences between bacterial species.

In summary, our study provides the first evidence of a role of the *Acod1* cis-aconitate decarboxylase enzyme producing itaconate in the pulmonary control of *Brucella* infection in a mouse model. We also demonstrate that this control is exerted at the cellular level, in alveolar macrophages, and acts via the bacterial enzyme isocitrate lyase. As the mouse ACOD1 enzyme is ∼80% identical in its amino acid sequence to the human ACOD1, with all five predicted cis-aconitate decarboxylase domains fully conserved [18], the development of pharmacological agents that enhance ACOD1 function or promote itaconate production might help to control early stages of pulmonary *Brucella* infection.

## Materials and methods

### Ethics statement

The procedures used in this study and the handling of the mice complied with current European legislation (Directive 86/609/EEC). The Animal Welfare Committee of the Université de Namur (UNamur, Belgium) reviewed and approved the complete protocol for *Brucella melitensis* and *B*. *abortus* infection (Permit Number: UN-LE-18/309).

### Mice, bacterial strains and reagents

Wild-type (wt) C57BL/6 mice were acquired from Harlan (Bicester, UK). *Acod*1^-/-^ C57BL/6 mice (49) were acquired from Dr. Eik Hoffmann (University of Lille, France). All wt and deficient mice used in this study were bred in the animal facility of the Gosselies campus of the Université Libre de Bruxelles (ULB, Belgium).

The wt *B*. *melitensis* 16M strain used here was a *B*. *melitensis* 16M stably expressing a rapidly maturing variant of the red fluorescent protein DsRed (55), the mCherry protein, under the control of the strong *Brucella* spp. promoter, p_sojA_. The construction of the mCherry-producing *Brucella* strain has been described previously in detail [37]. We also used mCherry-wt *B*. *abortus* 2308 [41] and mCherry-Δ*aceA* (isocitrate lyase) *B*. *abortus* [40] and the *Brucella abortus* 2308 Δ*aceA* strain complemented with the pMR10Ω*aceA* plasmid. Briefly, the *aceA* gene and its promoter were amplified with the Q5 High-Fidelity DNA polymerase (New England Biolabs) with the forward and reverse primers 5’-CGC GGATCC ATT TCC ACC AGT TCC TGA TC -3’ and 5’-AAAA CTGCAG GGA TTG TTC TTC TGC TCT TTC-3’. The PCR products were purified (Macherey-Nagel Clean-up kit) and cloned into the *EcoRV* site of the pGemT in *E*. *coli* DH10B. The *aceA* gene was then subcloned into the pMR10 plasmid (a kind gift from C.D. Mohr and R.C. Roberts, Stanford University) with the restriction enzymes *BamHI* and *PstI*. The resulting plasmid was introduced into *E*. *coli* strain S17.1 [42] and into *B*. *abortus* 2308 Δ*aceA* by mating. *Brucella* strains were always handled under Biosafety Level 3 containment according to Council Directive 98/81/EC of 26 October 1998 and a law of the Walloon government of 4 July 2002.

Itaconate (Sigma-Aldrich) and dimethyl itaconate (DMI) (Sigma-Aldrich) were diluted in bidistilled water, while 4-octyl itaconate (4OI) (Sigma-Aldrich) was diluted in DMSO. After filtration, the solutions were stored at 4°C.

### Murine Bone-Marrow Macrophages culture and infection

Murine Bone-Marrow Macrophages (BMDM) were obtained by sampling tibias and femur bones from 7- to 12-week-old wild type and *Acod1*^-/-^ C57BL/6 mice. BMDM were obtained by seeding 10^7^ bone marrow cells in 75 cm^2^ flasks in RPMI 1640 Glutamax medium (Gibco) supplemented with 10% heat-inactivated fetal bovine serum (FBS) (Gibco) and 10% L929 cell supernatant containing Macrophage Colony-Stimulating Factor (M-CSF). After 4 days incubation, the medium was changed and cells were put back in the incubator for 3 more days. The day before the infection, BMDM were seeded in 24-well plates at a concentration of 3.25x10^5^ cells per mL and left in the incubator overnight.

Infections were performed at a multiplicity of infection of 50:1 by centrifuging bacteria onto BMDM at 170 g for 10 min at 4°C, and then incubating the cells for 60 min at 37°C under 5% CO_2_ atmosphere. BMDM were extensively washed with PBS to remove extracellular bacteria and incubated for an additional 60 min in medium supplemented with either 50 μg/mL gentamicin to kill extracellular bacteria. One hour later, the medium was replaced by fresh medium supplemented with 10 μg/mL of gentamicin. At different times post infection, BMDM were scratched and lysed in PBS/0.1% X-100 Triton (Sigma-Aldrich) and CFU were plated onto 2YT agar plates using different dilutions to enumerate CFUs.

### Brucella infection in vivo

*Brucella* cultures were grown overnight with shaking at 37°C in 2YT liquid medium (Luria-Bertani broth with double quantity of yeast extract) and were washed twice in RPMI 1640 (Gibco Laboratories) (2000 xg, 10 min) before inoculation of the mice.

For intranasal infection, mice were anesthetized with a cocktail of Xylasine (9 mg/kg) and Ketamine (36 mg/kg) in PBS before being inoculated by intranasal injection with the indicated dose of *Brucella* in 30 μl of RPMI. Control animals were inoculated with the same volume of RPMI. The infectious doses were validated by plating serial dilutions of the inoculums. At the selected time after infection, mice were sacrificed by cervical dislocation. Immediately after sacrifice, the lungs and spleen were collected for bacterial count, flow cytometry, qRT-PCR, purification of the AMs and/or microscopic analyses.

For intraperitoneal infection, mice were injected with 500 μl intraperitoneally of the indicated dose of CFU without anesthesia.

### Bacterial counting

Organs were homogenized in PBS/0.1% X-100 Triton (Sigma-Aldrich). We performed successive serial dilutions in PBS to obtain the most accurate bacterial count and plated them on 2YT medium. The CFU were counted after 4 days of incubation at 37°C.

### RNA extraction

RNA from the whole lungs and spleen was extract by the Tripure/Chloroform method. Briefly, the lungs and spleen were harvested from mice, cut into small pieces and homogenized in 1 mL of Tripure (Tripure Isolation Reagent–Roche). After 5 min of incubation at RT, 200 μL of chloroform was added, and the tube was mixed vigorously for 15 seconds then incubated for 10 min at RT. After centrifugation for 15 min (12,000 xg at 4°C), the aqueous phase was collected and placed in a new tube. 500 μL of isopropanol was added. The tube was mixed by inversion and centrifuged for 10 min (12,000 xg at 4°C). The pellet was washed twice with 75% ethanol (7,500 xg for 5 min at 4°C), suspended in 50 μL of water, and incubated for 10 min at 55°C to completely resuspend the RNA pellet. RNA from purified AMs was extracted using the RNeasy Mini kit (Qiagen).

### RNA sequencing and analysis

After DNAse I, RNAse-free treatment (Thermo Scientific), the RNA samples were then transformed into cDNA, and the library was prepared. The Novaseq 6000 Trueseq SBS reagents (25 million paired-end reads) and Trueseq stranded RNA library preparation were used for the Illumina sequencing.

Genes with no raw read count were filtered out with an R script. Raw read counts were normalized and a differential expression analysis was performed with DESeq2 by applying an adjusted p-value < 0.05 and absolute log2-ratio > 0.5849.

### qRT-PCR analysis

RNA was treated with the DNAse I, RNAse-free kit (Thermo Scientific). Briefly, 2 μg of RNA was treated for 30 min with DNAse I at 37°C followed by DNAse I inactivation with 50 mM of EDTA for 10 min at 65°C. The RNA was then reverse transcribed with Superscript II reverse transcriptase (Invitrogen) with hexamer random primers as described by the manufacturer. A condition without reverse transcriptase was also conducted in parallel as a negative control. cDNA was then mixed with SybrGreen mix (FastStart universal SYBR Green Master (Rox); Roche) and the appropriate primer sets and subjected to qRT-PCR in a LightCycler 96 (Roche). The forward and reverse primers used were 5’-GGC ACA GAA GTG TTC CAT AAA GT-3’ and 5’-GAG GCA GGG CTT CCG ATA G-3’ for *Acod1*, 5’-GCG AAC AAA GCC AGA TGC AA-3’ and 5’-CCC CTT TCC TCC CAA ACC AA-3’ for *Tnf*, 5’-CGC ATG TTC CTG GGG AGA TT-3’ and 5’-TGG GAT GCA ACA TGG CTC TT-3’ for IL-1, 5’-GGC TTG CCC CAC TAC TTA GG-3’ and 5’-GCG AAC AAA GCC AGA TGC AA-3’ for IL-6. TATA binding protein (*Tbp*, forward 5’-GTT GGG GTG GCA TTT TCT GTG-3’, reverse 5’-GGC CTC TGC ATG TGT TCT CAT-3’) mRNA was used as the reference housekeeping gene for normalization. A total of 45 three-step cycles were performed as follows: 95°C for 10 sec, 60°C for 10 sec, and 92°C for 10 sec. Melting curves were then performed to assess primer specificity. The target mRNA fold change was calculated based on the 2^-ΔΔCt^ formula, where the *Tbp* gene was used as the reference gene, and RNA from naïve mice was used as the standard condition. At least 6 biological replicates and 3 technical replicates were performed for each gene tested.

### Bioscreen analysis

Overnight cultures were prepared in 2YT rich medium the day before in order to obtain an OD_600 nm_ between 0.2–0.5 the day after. Cultures were washed twice in PBS (2000 xg for 10 min at RT), and then suspended in 2 YT rich medium or Plommet-Erythritol minimal medium [30] to obtain an OD_600 nm_ = 0.05 in a final volume of 700 μL. We used the Bioscreen system (Thermo Fisher) to measure the growth of *Brucella* at 37°C for 72 hours.

### Brucella melitensis staining with eFluor^670^

For some histological and flow cytometry experiments, we stained *B*. *melitensis* with eFluor^670^ labelling. Cultures (10 mL) were grown overnight as indicated above, bacteria from 1 mL of culture were centrifuged (2 min, 7500 xg, RT) and the pellets were washed 3 times with 1 mL of PBS. Then, the bacteria were incubated for 20 min at RT in the dark with eFluor^670^ dye at the final concentration of 10 μM in 1 mL of PBS. After incubation, the bacteria were washed three times in 1 mL of PBS and diluted once in PBS to obtain the precise infectious dose before inoculation of the mice.

### Cytofluorometric analysis

The lungs were harvested and cut into small pieces and incubated for 1 hour at 37°C with a mix of 100 μg/mL of DNAse I fraction IX (Sigma-Aldrich) and 1.6 mg/mL of collagenase (400 Mandl U/mL), as described previously (6). The cells were then washed, filtered and incubated with saturating doses of purified 2.4G2 (anti-mouse Fc receptor, ATCC) in 200 μl PBS, 0.2% BSA, 0.02% NaN3 (FACS buffer) for 20 min at 4°C to prevent antibody (Ab) binding to the Fc receptor. 3–5x10^6^ cells were stained on ice with various fluorescent mAb combinations in FACS buffer: BV650-coupled RA3-6B2 (anti-B220, BD Horizon), FITC-coupled RA3-6B2 (anti-B220, BD Pharmingen), Alexa Fluor 700-coupled M1/70 (anti-CD11b, BD Pharmingen), BV421-coupled N418 (anti-CD11c, BD Horizon), BV480-coupled P84 (anti-CD172a, BD OptiBuild), BV711-coupled 1D3 (anti-CD19, BD Horizon), FITC-coupled 1D3 (anti-CD19, BD Pharmingen), BV786-coupled H194-112 (anti-CD26, BD OptiBuild), FITC-coupled 145-2C11 (anti-CD3e, BD Pharmingen), Pacific Blue-coupled 500A2 (anti-CD3e, BD Horizon), Alexa Fluor 700-coupled RM4-5 (anti-CD4, BD Pharmingen), PE-CF594-coupled 30-F11 (anti-CD45, BD Horizon), APC-coupled 53–7.3 (anti-CD5, BD Pharmingen), BV650-coupled X45-5/7.1 (anti-CD64, BD OptiBuild), APC-H7-coupled 53–6.7 (anti-CD8α, BD Pharmingen), FITC-coupled BM8 (anti-F4/80, eBiosciences), BV711-coupled M5/114.15.2 (anti-I-A/I-E, BD Horizon), PerCP-Cy5.5-coupled HK1.4 (anti-Ly6C, BioLegend), APC-H7-coupled 1A8 (anti-Ly6G, BD Pharmingen), FITC-coupled PK136 (anti-NK1.1, BD Pharmingen), PE-coupled PK136 (anti-NK1.1, BD Pharmingen), PE-coupled E50-2440 (anti-Siglec F, BD Pharmingen), FITC-coupled GL3 (anti-TCR γδ, BD Pharmingen), APC-coupled ZET (anti-XCR1, BioLegend). The cells were analyzed on a CytoFLEX LX (Beckman Coulter, 6 lasers) flow cytometer.

### Immunofluorescence microscopy of the lungs

Lungs were fixed for 20 minutes at RT in 2% paraformaldehyde (PFA). Then, still in 2% PFA, they were placed under a vacuum until no air was present in the lungs for 2 hours. The purpose of fixation is to kill *Brucella* so that the tissues can then be manipulated outside the biosafety level 3 laboratory. After PFA fixation and two washes in PBS, the lungs were incubated overnight at 4°C in a 20% PBS-sucrose solution. The tissues were then embedded in Tissue-Tek OCT compound (Sakura), frozen in liquid nitrogen, and cryostat sections (5 μm) were prepared. For staining, the tissue sections were rehydrated in PBS and incubated in a PBS solution containing 1% blocking reagent (Boeringer) (PBS-BR 1%) for 20 min before incubation overnight in PBS-BR 1% containing the following: the DAPI nucleic acid stain Alexa Fluor 350, 488 phalloidin (Molecular Probes) and Alexa Fluor 647–coupled RB6-8C5 anti-Ly6G/Ly6C (Gr1) (Biolegend). Slides were mounted in Fluoro-Gel medium (Electron Microscopy Sciences, Hatfield, PA). Labelled tissue sections were visualized with an Axiovert M200 inverted microscope (Zeiss, Iena, Germany) equipped with a high-resolution monochrome camera (AxioCam HR, Zeiss). Images (1384x1036 pixels, 0.16 μm/pixel) were acquired sequentially for each fluorochrome with A-Plan 10x/0.25 N.A. and LD-Plan-NeoFluar 63x/0.75 N.A. dry objectives and recorded as eight-bit grey-level *.zvi files. At least 3 slides were analyzed per organ from 3 different animals and the results are representative of 2 independent experiments.

### Purification and transmission electron microcopy analysis of alveolar macrophages

Pulmonary alveolar macrophages were obtained by homogenization and filtration of lungs, density gradient centrifugation (5 mL of 1.085 g/cm^3^ nycodenz were mixed with the cells, then 5 mL of cold RPMI was added gently above the nycodenz, and the tube was centrifuged at 1700 x g for 30 min, with minimum braking). CD11c-specific Magnetic associated cell sorting (MACS) was performed on the low-density cells collected at the interface between the nycodenz and RPMI using MiniMACS, MS column and CD11c+ magnetic beads (Miltenyl Biotec).

Purified alveolar macrophages were fixed for 2 hours in 2.5% glutaraldehyde in 0.1 M cacodylate buffer at 4°C, washed 3 times (4000 xg, 5 min) with 0.2 M cacodylate buffer then postfixed in 2% osmium tetroxide in 0.1 M cacodylate buffer for 1 hour at RT. After 3 washes, samples were serially dehydrated in ethanol (30% EtOH first for 5 min, followed by 10 min–the same for 50%, 70%, 85%, 100% EtOH). Propylene oxide was then added 4x5 min at RT and the pellet was progressively embedded in epoxy resin (Agar 100 resin; Agar Scientific, United Kingdom) (75:25, 50:50 and then 25:75% of propylene oxide/resin). Ultrathin 50-nm sections were obtained, mounted on copper-Formvar-carbon grids (EMS, United Kingdom), and stained with uranyl acetate and lead citrate by standard procedures. Observations were made on a Tecnai 10 electron microscope (FEI, Eindhoven, The Netherlands), and images were captured with a Veleta charge-coupled-device (CCD) camera and processed using the AnalySIS and Adobe Photoshop software programs.

### Structural analysis

The docking of itaconate into the catalytic site of isocitrate lyase from *Brucella abortus* was performed using Maestro 11.9 from Schrodinger’s suite 2019. The starting crystal structure corresponds to a complex of the enzyme with malonate (chain A of 3OQ8 PDB entry). The itaconate ligand (doubly deprotonate dicarboxylate form) was build based on the malonate from this PDB structure (using “3D Builder” tool). The resulting complex was further minimized using the “Protein Preparation Wizard” [43]. Interactions were analyzed with Maestro 11.9 and visualized with the software Pymol (version 1.7.4.4; Schrödinger).

A crystal structure of *Brucella abortus* isocitrate lyase in complex with itaconate has also been obtained at 1.8 Å resolution. Statistics of data collection and refinement together with coordinates have been deposited at the PDB (entry 7RBX).

### Statistical analysis

We used a (Wilcoxon-)Mann-Whitney test provided by the GraphPad Prism software to statistically analyze our results. Each group of deficient mice was compared to the wt mice. We also compared each group with the other groups and displayed the results when required. Values of p < 0.05 were considered to represent a significant difference. *, **, *** denote p < 0.05, p < 0.01, p< 0.001 respectively.

## Supporting information

S1 Fig Alveolar macrophages are the main *B*. *melitensis* infected cells in lungs. Wild-type C57BL/6 mice (n = 5) received PBS intranasally (control mice) or 10^7^ CFU of mCherry-expressing *B*. *melitensis* labelled with eFluor^670^. Mice were sacrificed at 24 hours post-infection. The lungs were harvested, and the cells were isolated and then analyzed by flow cytometry for the FSC and the expression of eFluor^670^, mCherry, CD11c, and Siglec-F as indicated. (**A)** Gating strategy. Numbers indicate the percentage of eFluor^670+^ cells among the total cells (upper panels) and the percentage of eFluor670^+^ cells that are also positive for CD11c and Siglec-F markers (lower panels) in naïve mice (left-hand panels) or infected mice (right-hand panels). **B.** Same gating strategies, but with previously purified alveolar macrophages. These results are representative of three independent experiments.
(TIF)

S2 Fig *Acod1* gene deficiency does not affect *does not affect splenomegaly induced following Brucella infection*. Wild-type and *Acod1*^-/-^ C57BL/6 mice were intranasally infected with 10^7^ CFU of wild-type *B*. *melitensis* 16M or *B*. *abortus* 2308, as indicated. At 2-, 5-, 9- and 28-days post-infection, spleen were harvested and weighed individually. Each point represents one mouse, n = 8. Grey bar represents the mean. ns = non-significant differences between the indicated groups. Data are representative of 2 independent experiments.
(TIF)

S3 Fig *Acod1* gene deficiency does not affect *B*. *abortus* control in the spleen from intraperitoneally infected mice. Wild-type and *Acod1*^-/-^ C57BL/6 mice were intraperitoneally infected with 10^7^ CFU of wild-type *B*. *abortus* 2308, as indicated. At 2-, 5- and 9-days post-infection, spleen were harvested and CFU were counted. Each point represents one mouse, n = 8. Grey bar represents the mean. ns = non-significant differences between the indicated groups. Data are representative of 2 independent experiments.
(TIF)

S4 Fig Gating strategy of flow cytometry analysis. Representative picture of the gating strategies used for the discrimination of alveolar macrophages (AM), classical dendritic cells, monocytes, neutrophils, eosinophils, B cells, CD4+T cells, CD8+T cells, γδT cells, NK T cells and NK cells among lung cells from naive wild type C57BL/6 mice analyzed using CytoFLEX flow cytometer (Beckman Coulter, 6 lasers). “Lineage” staining groups together the markers CD3, CD19, B220 and NK1.1. The numbers indicate the percentages in each quadrant.
(TIF)

S5 Fig 4-octyl itaconate inhibits *Brucella* growth *in vitro*. Comparison of the impact of different concentrations of 4-octyl itaconate on the growth of wild-type *B*. *melitensis* or *B*. *abortus* in rich medium (2YT). The bacteria were grown for 72 hours at 37°C and the OD was measured every 30 min in a Bioscreen system. The standard deviation was obtained from three independent experiments.
(TIF)

S6 Fig Dimethyl itaconate is bactericidal for *B*. *abortus*. A pre-culture of *B*. *abortus* was prepared in 2YT and was then washed and diluted 10 times in Plommet Erythritol medium supplemented (10 mM DMI) or not (ctl) with 10 mM of DMI. The cultures have grown at 37°C overnight. The day after, cultures were washed twice in PBS and plated on 2YT using serial dilutions to obtain a countable number of CFU. Experiment was repeated 2 times, in triplicates. Significant difference between the two groups is marked with asterisks: ***p < 0.001.
(TIF)

S7 Fig Dimethyl itaconate impacts wild-type and *ΔaceA B*. *abortus* morphology *in vitro*. Transmission electronic microscopy images were performed as described in the Materials and Methods section on wild-type and Δ*aceA B*. *abortus*, cultured in poor medium (Plommet-Erythritol) overnight supplemented or not with 1 mM of dimethyl itaconate. Red arrows indicate membrane alterations.
(TIF)

S1 Data RNAseq of whole lungs from infected versus naïve mice. Wild-type C57BL/6 mice intranasally infected with 10^7^ CFU of wild-type *B*. *melitensis* (n = 3), or receiving the same volume of PBS (naïve group) (n = 3), were sacrificed at 24 hours post-infection. Lungs were harvested and RNA was extracted. RNAseq was performed with the Illumina system and Deseq2 analysis as described in the Materials and Methods. Data represent the list of genes associated with their fold change (FC) (Log_2_) and adjusted p-value—log10 (false discovery rate, FDR). Data are representative of two independent experiments. These data have been deposited in the GEO database under the reference GSE180699.
(XLSX)

S2 Data RNAseq of alveolar macrophages from infected versus naïve mice. Wild-type C57BL/6 mice intranasally infected with 10^7^ CFU of wild-type *B*. *melitensis* (n = 3), or receiving the same volume of PBS (naïve group) (n = 3), were sacrificed at 24 hours post-infection. Lungs were harvested, alveolar macrophages were purified and RNA was extracted. RNAseq was performed with the Illumina system and Deseq2 analysis as described in the Materials and Methods. Data represent the list of genes associated with their fold change (FC) (Log_2_) and adjusted p-value—log10 (false discovery rate, FDR). Data are representative of two independent experiments. These data have been deposited in the GEO database under the reference GSE180699.
(XLSX)
