# Supplementary figures and images for: Aconitate decarboxylase 1 participates in the control of pulmonary Brucella infection in mice
Source: PLoS Pathog. 2021 Sep 15;17(9):e1009887. doi: 10.1371/journal.ppat.1009887 (PMC8443048; doi:10.1371/journal.ppat.1009887)

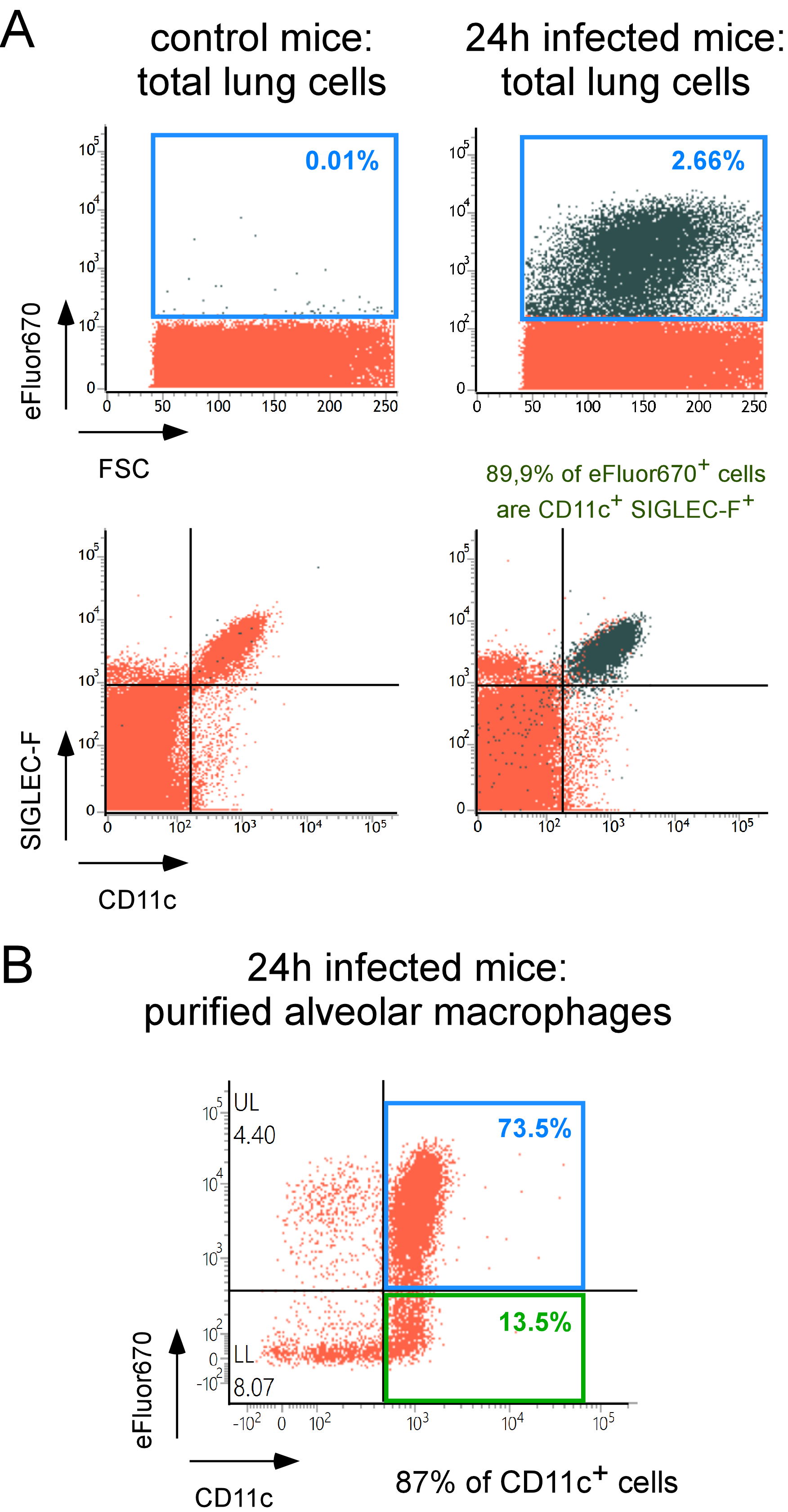

Supplement: S1 Fig — Wild-type C57BL/6 mice (n = 5) received PBS intranasally (control mice) or 107 CFU of mCherry-expressing B. melitensis labelled with eFluor670. Mice were sacrificed at 24 hours post-infection. The lungs were harvested, and the cells were isolated and then analyzed by flow cytometry for the FSC and the expression of eFluor670, mCherry, CD11c, and Siglec-F as indicated. (A) Gating strategy. Numbers indicate the percentage of eFluor670+ cells among the total cells (upper panels) and the percentage of eFluor670+ cells that are also positive for CD11c and Siglec-F markers (lower panels) in naïve mice (left-hand panels) or infected mice (right-hand panels). B. Same gating strategies, but with previously purified alveolar macrophages. These results are representative of three independent experiments. (TIF) [file ppat.1009887.s001.tif]

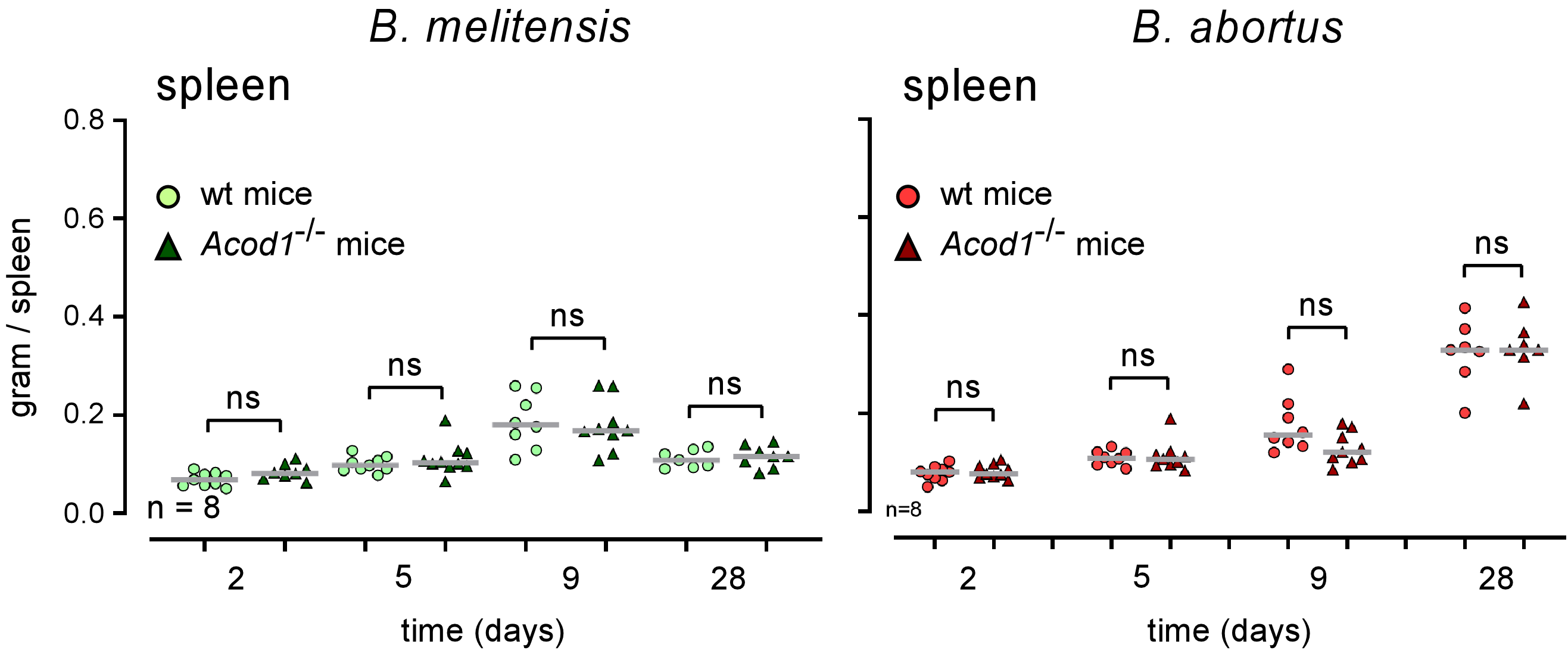

Supplement: S2 Fig — Wild-type and Acod1-/- C57BL/6 mice were intranasally infected with 107 CFU of wild-type B. melitensis 16M or B. abortus 2308, as indicated. At 2-, 5-, 9- and 28-days post-infection, spleen were harvested and weighed individually. Each point represents one mouse, n = 8. Grey bar represents the mean. ns = non-significant differences between the indicated groups. Data are representative of 2 independent experiments. (TIF) [file ppat.1009887.s002.tif]

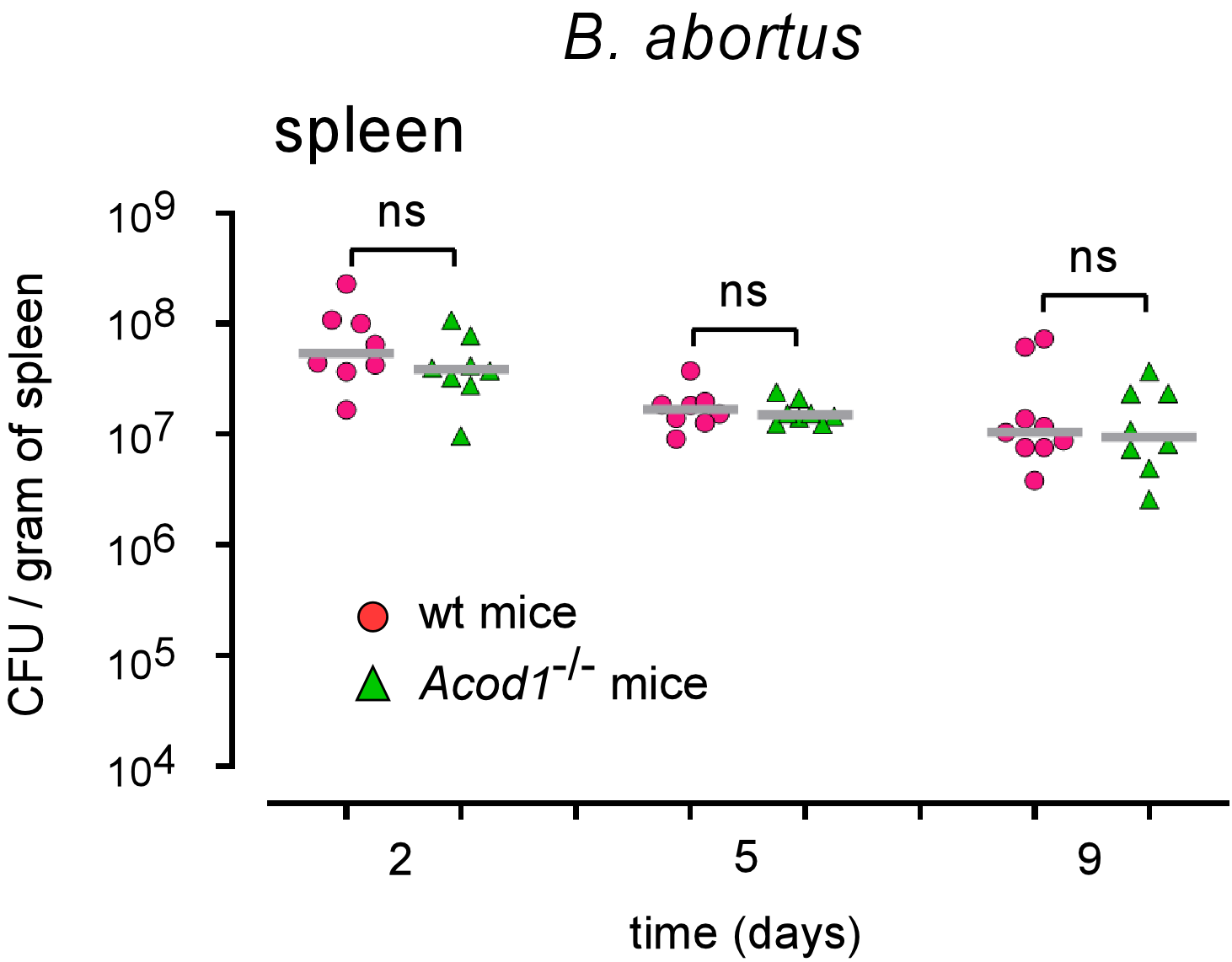

Supplement: S3 Fig — Wild-type and Acod1-/- C57BL/6 mice were intraperitoneally infected with 107 CFU of wild-type B. abortus 2308, as indicated. At 2-, 5- and 9-days post-infection, spleen were harvested and CFU were counted. Each point represents one mouse, n = 8. Grey bar represents the mean. ns = non-significant differences between the indicated groups. Data are representative of 2 independent experiments. (TIF) [file ppat.1009887.s003.tif]

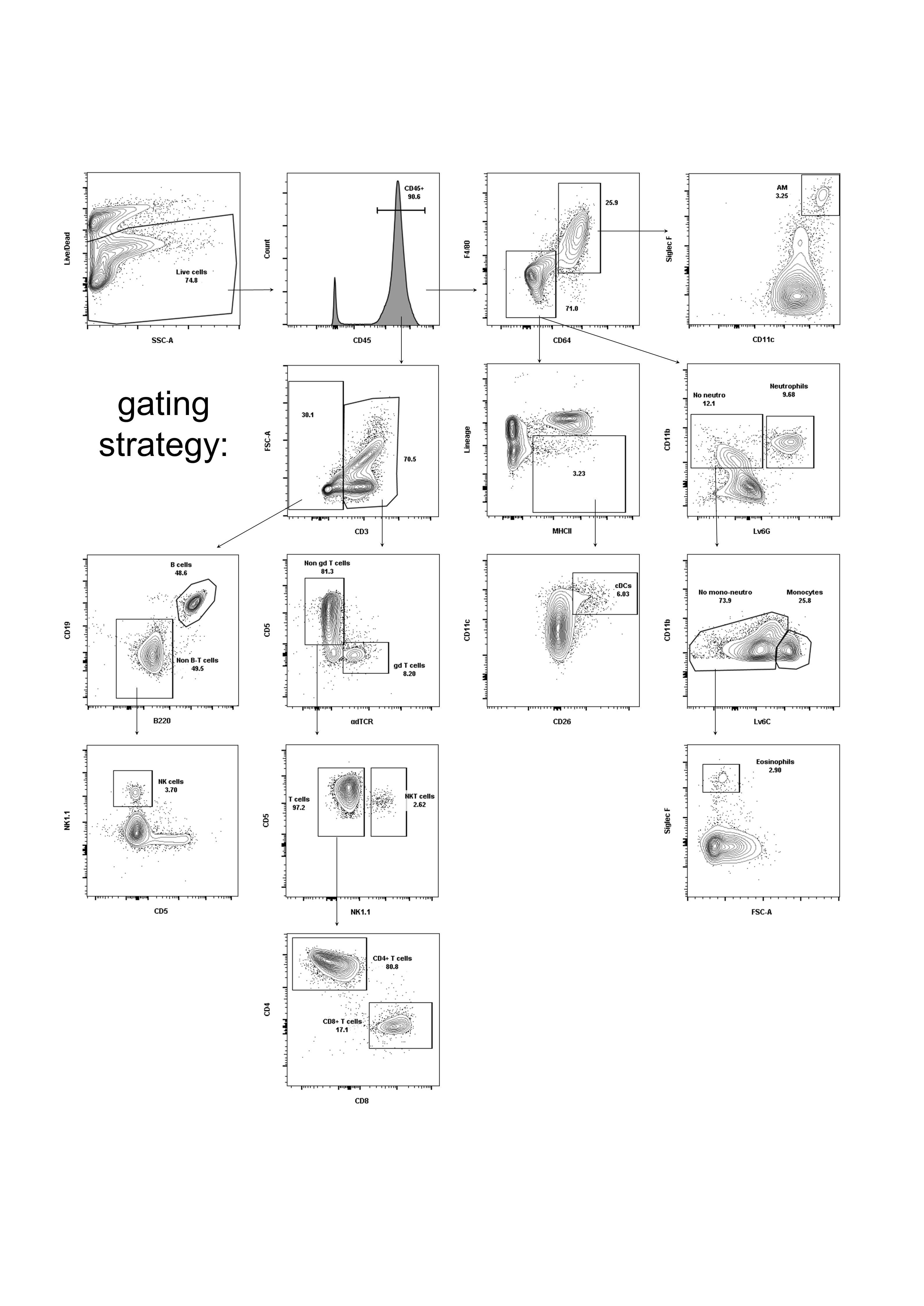

Supplement: S4 Fig — Representative picture of the gating strategies used for the discrimination of alveolar macrophages (AM), classical dendritic cells, monocytes, neutrophils, eosinophils, B cells, CD4+T cells, CD8+T cells, γδT cells, NK T cells and NK cells among lung cells from naive wild type C57BL/6 mice analyzed using CytoFLEX flow cytometer (Beckman Coulter, 6 lasers). “Lineage” staining groups together the markers CD3, CD19, B220 and NK1.1. The numbers indicate the percentages in each quadrant. (TIF) [file ppat.1009887.s004.tif]

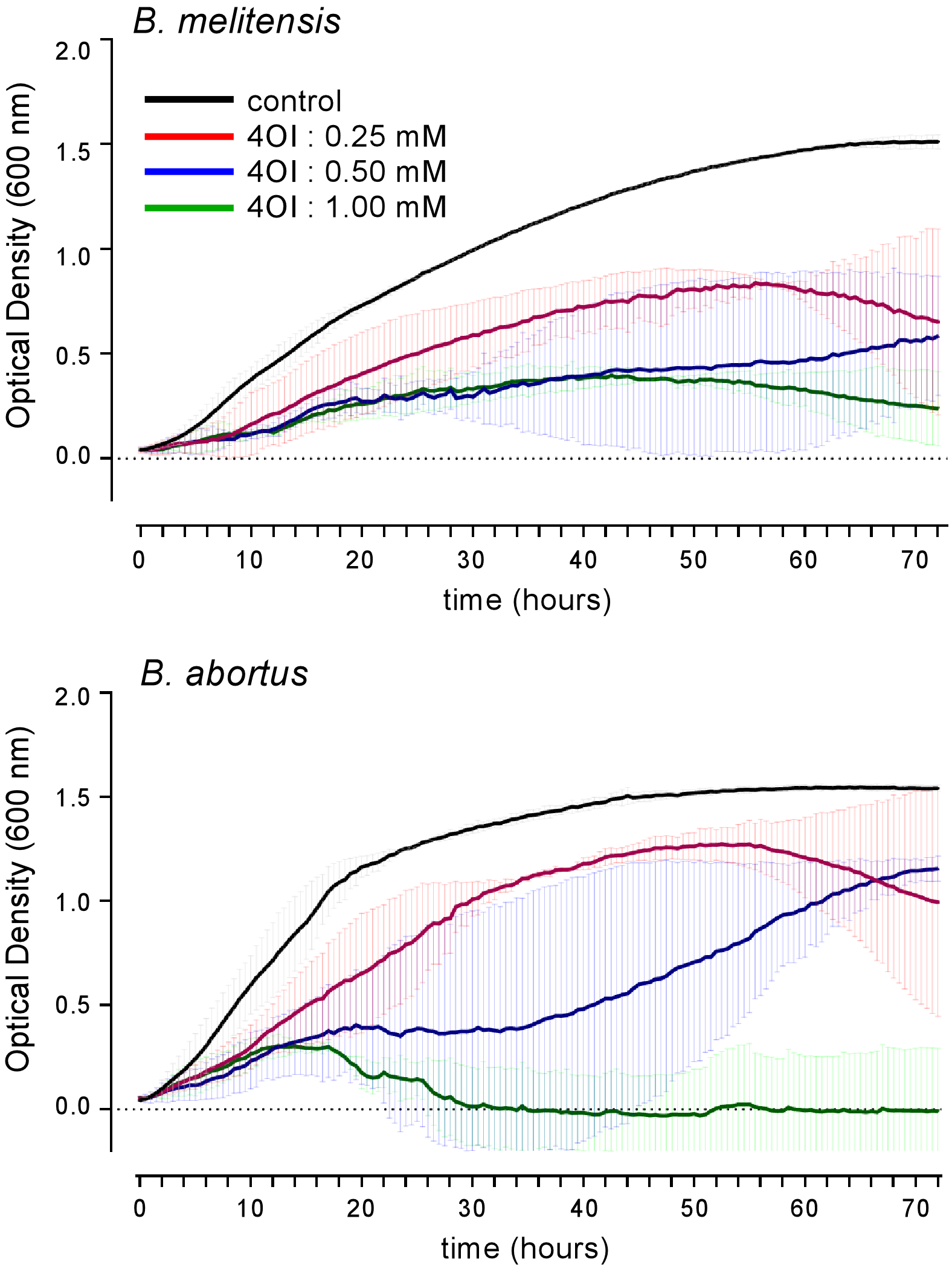

Supplement: S5 Fig — Comparison of the impact of different concentrations of 4-octyl itaconate on the growth of wild-type B. melitensis or B. abortus in rich medium (2YT). The bacteria were grown for 72 hours at 37°C and the OD was measured every 30 min in a Bioscreen system. The standard deviation was obtained from three independent experiments. (TIF) [file ppat.1009887.s005.tif]

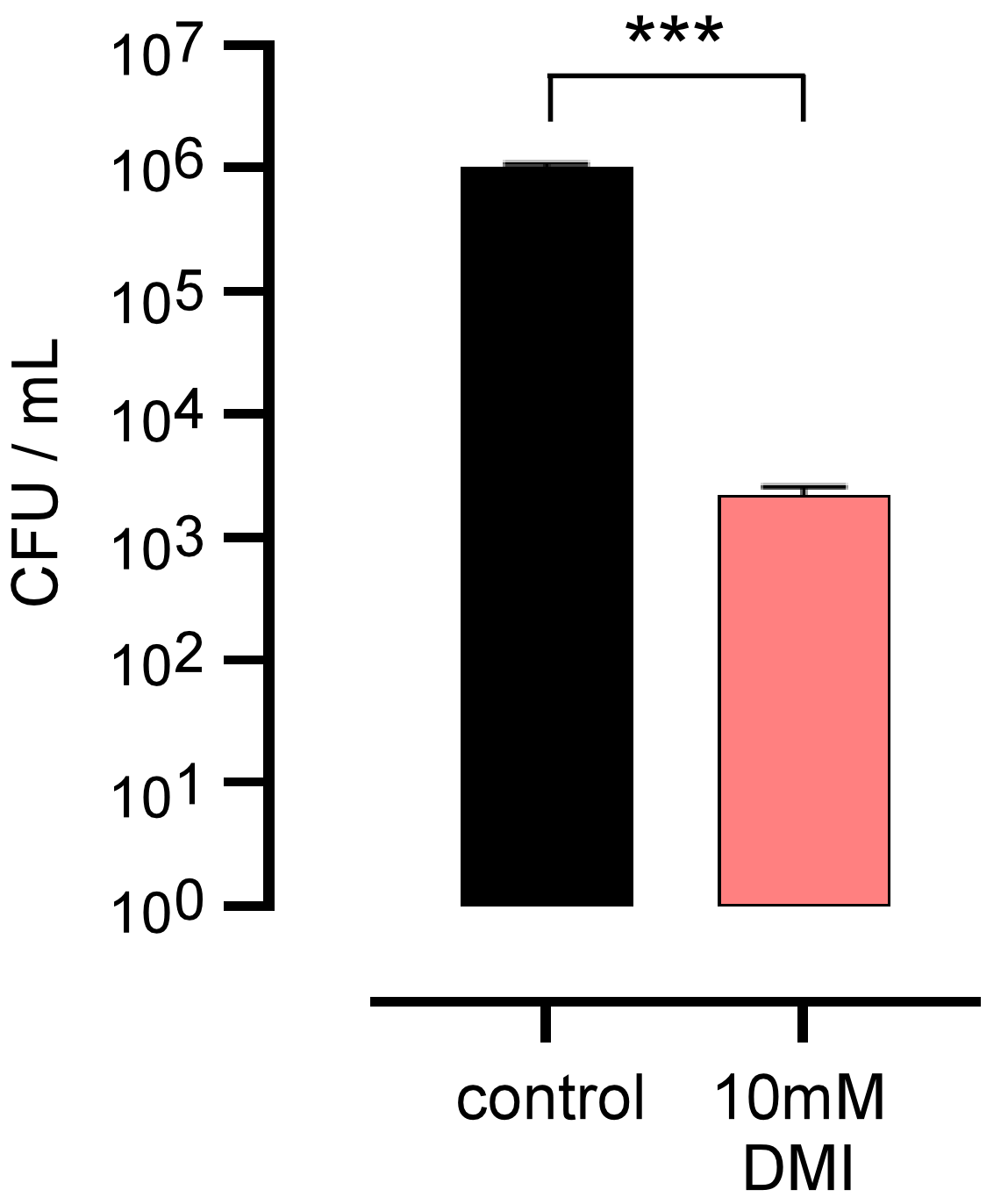

Supplement: S6 Fig — A pre-culture of B. abortus was prepared in 2YT and was then washed and diluted 10 times in Plommet Erythritol medium supplemented (10 mM DMI) or not (ctl) with 10 mM of DMI. The cultures have grown at 37°C overnight. The day after, cultures were washed twice in PBS and plated on 2YT using serial dilutions to obtain a countable number of CFU. Experiment was repeated 2 times, in triplicates. Significant difference between the two groups is marked with asterisks: ***p < 0.001. (TIF) [file ppat.1009887.s006.tif]

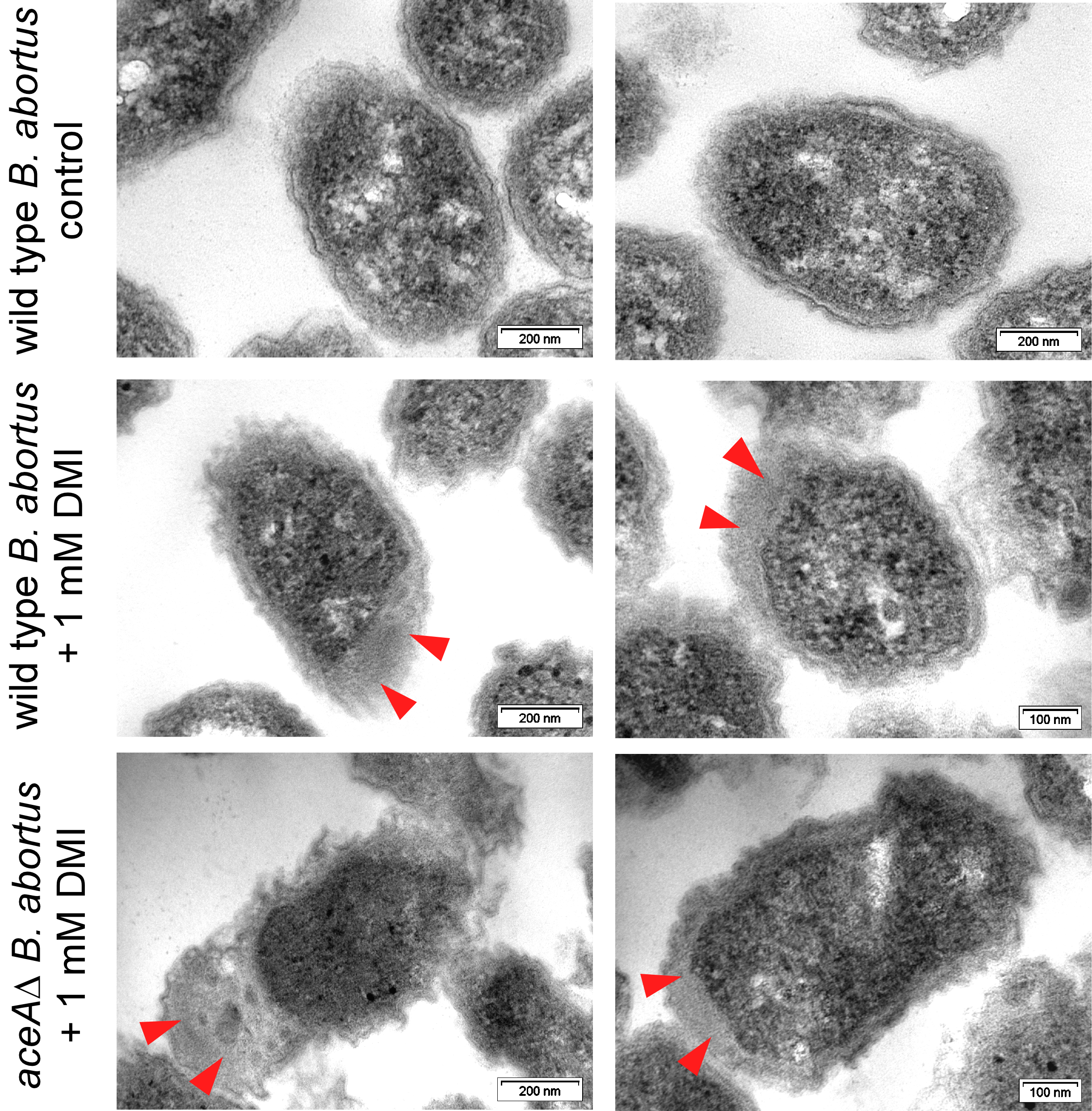

Supplement: S7 Fig — Transmission electronic microscopy images were performed as described in the Materials and Methods section on wild-type and ΔaceA B. abortus, cultured in poor medium (Plommet-Erythritol) overnight supplemented or not with 1 mM of dimethyl itaconate. Red arrows indicate membrane alterations. (TIF) [file ppat.1009887.s007.tif]
